# T- and pH-Dependent Hydroxyl-Radical Reaction Kinetics of Lactic Acid, Glyceric Acid, and Methylmalonic Acid in the Aqueous Phase

**DOI:** 10.1021/acs.jpca.4c08063

**Published:** 2025-02-14

**Authors:** Yuehuan Hu, Yimu Zhang, Liang Wen, Thomas Schaefer, Hartmut Herrmann

**Affiliations:** † School of Environmental Science and Engineering, Shandong University, Qingdao 266237, China; ‡ Atmospheric Chemistry Department (ACD), Leibniz-Institute for Tropospheric Research (TROPOS), Permoserstraße 15, 04318 Leipzig, Germany

## Abstract

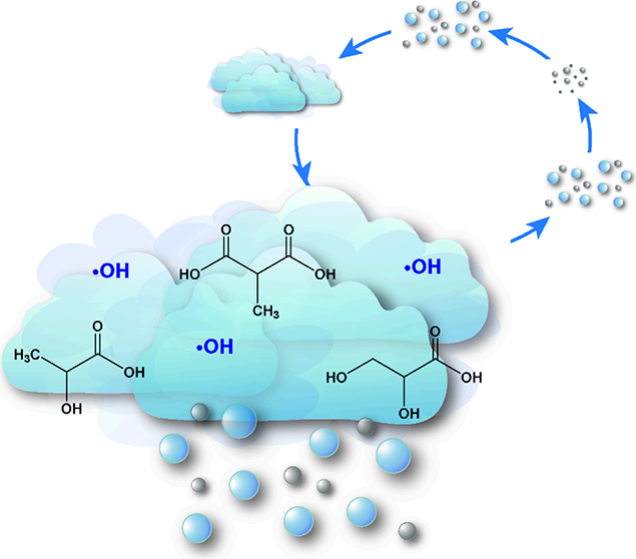

Carboxylic acids are a common class of compounds found in atmospheric aerosols and cloud droplets. In this study, the oxidation kinetics of several carboxylic acids in the aqueous phase by the atmospherically relevant ^•^OH radical were investigated to better understand the loss processes for this class of compounds. The rate constants for the reactions of the ^•^OH radical were determined using the thiocyanate competition kinetics method for lactic acid, glyceric acid, and methylmalonic acid as a function of temperature and pH. The Arrhenius equations for oxidation by the ^•^OH radical are as follows (unit in L mol^–1^ s^–1^): For lactic acid: *k*(*T*, HA) = (1.3 ± 0.1) × 10^10^ × exp[(−910 ± 160 K)/*T*] and *k*(*T*, A^–^) = (1.3 ± 0.1) × 10^10^ × exp[(−800 ± 80 K)/*T*]; for glyceric acid: *k*(*T*, HA) = (6.0 ± 0.2) × 10^10^ × exp[(−1100 ± 170 K)/*T*] and *k*(*T*, HA^±^) = (3.6 ± 0.1) × 10^10^ × exp[(−1500 ± 100 K)/*T*]; and for methylmalonic acid: *k*(*T*, H_2_A) = (5.5 ± 0.1) × 10^10^ × exp[(−1760 ± 100 K)/*T*], *k*(*T*, HA^–^) = (1.4 ± 0.1) × 10^9^ × exp[(−530 ± 80 K)/*T*] and *k*(*T*, A^2–^) = (9.6 ± 0.4) × 10^10^ × exp[(−1530 ± 270 K)/*T*]. The general trend of the ^•^OH rate constant was observed *k*_A^2–^_*> k*_HA^–^_*> k*_H_2_A_. The energy barriers of the ^•^OH radical reaction and thus the most probable site of H atom abstraction were calculated using density functional theory simulations in *Gaussian* with the M06-2X method and the 6-311++G(3df,2p) basis set. Kinetic data predicted from structure–activity relationships were compared to the measured ^•^OH radical rate constants. ^•^OH radical oxidation in the aqueous phase could serve as an important sink for carboxylic acids, and the pH- and T-dependent rate constants of ^•^OH radical reactions provide a better description of the aqueous-phase sink processes. Hence, the atmospheric lifetime as well as the partitioning of the investigated carboxylic acids was calculated.

## Introduction

Monocarboxylic acids (MCAs) and dicarboxylic acids (DCAs) are widely distributed in marine and continental aerosols and cloud droplets.^1−4^ Organic acid sources are either oxidation reactions of volatile organic compounds (VOCs) formed in the gas phase, or they are released directly into the atmosphere by anthropogenic emissions (e.g., biomass burning, industrial activities, and vehicle exhaust) or biogenic emissions (e.g., bacteria and plants).^5^ This compound class can influence the hygroscopicity of aerosols and the pH of fog, cloud droplets, and aerosols.^2,4^ In accretion reactions, acids can contribute to the formation of oligomers in deliquescent aerosols.^6,7^ Additionally, organic acids are able to affect metal complexation and thus photochemistry in aerosols and droplets. In general, the oxidation of organic acids in the aqueous phase leads to more oxidized compounds, as well as a shortening of the carbon chain. Based on the acid–base equilibrium, organic acids exist in different protonation states. The pH of the atmospheric aqueous phase varies from strongly acidic to neutral conditions.^6^ Under strongly acidic conditions, the organic acids are fully protonated (H_2_A/HA), and under neutral conditions to weakly alkaline conditions, they are fully deprotonated (A^2–^/A^–^).^6^ Since the protonated acids in the aqueous phase are both more volatile and less reactive with radicals than their deprotonated forms, the efficiency of the sink in the aqueous phase is strongly pH-dependent.^2^ To illustrate the importance of ^•^OH reactions in the aqueous phase, the partition ratio of carboxylic acids between the gas and aqueous phases can be described as follows. Using estimated Henry’s law constants for lactic acid, glyceric acid, and methylmalonic acid of 8.9 × 10^3^ mol l^–1^ atm^–1^, 2.4 × 10^5^ mol l^–1^ atm^–1^, 1.9 × 10^8^ mol l^–1^ atm^–1^,^8^ and a liquid water content (LWC) of 1 × 10^–5^ to 1 g m^–3^ used from previous aerosol and cloud measurements,^6,7^ a partition ratio in the range of 10^–13^ - 10^–8^ at 298 K is obtained. This indicates that under aerosol conditions (lower LWC range) only methylmalonic acid is present, while under cloud conditions (higher LWC range) all three carboxylic acids: lactic acid, glyceric acid, and methylmalonic acid, are present in the aqueous phase, based on the calculated Henry’s solubilities.

Thus, to better understand the initial steps of aerosol particle processing or “aging”, studies of the reaction kinetics of MCA and DCA in the aqueous phase are required. The main oxidant in the aqueous phase of the atmosphere is the ^•^OH radical, which is formed mainly by the Fenton reaction and photolysis of H_2_O_2_ or enters the aqueous phase by uptake from the gas phase.

Within the present study, the thiocyanate competition kinetics method was used to investigate the ^•^OH radical constants of lactic acid, glyceric acid, and methylmalonic acid. The laser flash photolysis - long path absorption (LFP-LPA) technique was used to generate the ^•^OH radicals and detect the reference compound.

The rate constants of the different protonation forms (H_2_A, HA^–^, and A^2–^) of the respective carboxylic acids of the ^•^OH radical reaction were determined under strongly acidic, slightly acidic, and slightly alkaline conditions to represent all three forms for each of the acids. The measurements were conducted over a temperature range from 278 to 318 K, with 10 K interval, to cover the typical temperature range of liquid water in the lower troposphere. Arrhenius expressions were derived from the data to describe the *T*-dependences of the reactions and to enable the calculation of atmospheric lifetimes of the measured carboxylic acids. These lifetimes provide insights into the persistence of these compounds under varying atmospheric conditions. These findings contribute to the expansion of the kinetic database for atmospherically relevant aqueous-phase reactions and facilitate the improvement of structure–activity relationship (SAR) methods for kinetic predictions. This in turn improves the predictive capabilities of atmospheric chemistry models such as CAPRAM 4.0^9^ for atmospheric aqueous-phase reactions.

## Experimental Methods

### Experimental Setup for Kinetic Experiments

The laser flash photolysis-laser long-path absorption (LFP-LPA) experimental setup has been used to determine rate constants for radical reactions of specific organic compounds in the aqueous phase as described in earlier studies from our laboratory.^10−14^

In short, the main component of the LFP-LPA system is the temperature-controlled measuring cell (3.5 × 4 × 2 cm) equipped with high-purity SUPRASIL windows. The photochemical reactions were initiated by an excimer laser pulse (Compex 201, Lambda Physics). The wavelength of the pulse was λ = 248 nm, and the pulse frequency was *f* = 4 Hz. To monitor changes in light intensity due to radical reactions, a continuous-wave laser (LCX-561, Oxxius) or, alternatively, the (Model Radius 405, Coherent) with a wavelength of λ = 561 nm or, respectively, of λ = 407 nm was used in conjunction with a photodiode (S1336–44BQ, Hamamatsu) to observe the (SCN)_2_·^–^ absorption. To effectively increase the light path length through the measurement cell to *d* = 48 cm and to improve the sensitivity and accuracy of intensity measurements in solution, a white cell mirror configuration was used. Data was collected using an oscilloscope (Data SYS 944, Gould) connected to a computer used to store and analyze the data. A time profile averaged from 256 individual traces was used to determine the maximum absorbance. The traces were recorded for each concentration of the organic reactant in the aqueous solution.

The solution was supplied to the cell from a *T*-controlled reservoir, while the temperature of the reservoir was controlled from 278 to 318 K by a thermostatic controller (RC-6CS, Lauda).

### Chemicals and Other Equipment

The results of this study were obtained with the following chemicals used without further purification: lactic acid (C_3_H_6_O_3_, ≥99% Sigma-Aldrich), glyceric acid (C_3_H_6_O_4_, ≥99%, Sigma-Aldrich), methylmalonic acid (C_4_H_4_O_3_, ≥99%, Sigma-Aldrich), hydrogen peroxide (H_2_O_2_, ≥30% in water, Chemsolute), potassium thiocyanate (KSCN, ≥99%, Chemsolute), perchloric acid (HClO_4_, 70–72% in water, J. T. Baker Analyzed), and sodium hydroxide (NaOH, ∼1 mol L^–1^ in water, Fluka).

Pure water (18 MΩ cm) from a Milli-Q system (Millipore, Billerica, MA) was used for the preparation of all solutions. To measure the molar absorption coefficients (ε) and to verify the concentrations of the stock solutions, a dual-beam spectrometer (Lambda 900, PerkinElmer) was employed. A pH meter (Lab855, Schott) was used to determine the pH of the solutions.

### Hydroxyl Radical (^•^OH) Kinetics

As the absorption of the ^•^OH radicals is relativly weak and the absorptions of the subsequently formed alkyl and peroxyl radicals overlap, the competition kinetics method was used to determine the radical rate constants in the aqueous phase. The precursor solution was prepared with a concentration of 2 × 10^–4^ mol L^–1^ hydrogen peroxide (H_2_O_2_) as ^•^OH radical precursor and 2 × 10^–5^ mol L^–1^ potassium thiocyanate (KSCN) as ^•^OH radical scavenger. The ^•^OH radicals were generated at a concentration of approximately 10^–7^ mol L^–1^ via the photolysis of the precursor solution at a wavelength of λ = 248 nm. The concentrations of the organic reactants were gradually increased to 6.0 × 10^–4^ mol L^–1^ in the five different solutions of a series of measurements, for a rate constant. Two radical reactions take place in parallel, in which the photolytically generated ^•^OH radicals react either with the organic compound (RH) in (R-1) or with the reference compound in (R-2).
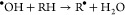
R-1
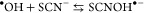
R-2
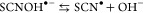
R-3
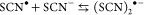
R-4
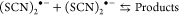
R-5

Absorption of the dithiocyanate radical anion ((SCN)_2_·^–^) formed in the reactions (R-2 - R-4) occurs in the visible range (λ = 400–600 nm)^15^ and is related to the ^•^OH concentration. The second-order rate constants were calculated according to Schaefer and Herrmann using (eq 1).^10^
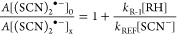
1

If the solution does not contain an organic reactant, A[(SCN)_2_^•–^]_0_ is the absorption maximum at λ = 561 nm or, respectively, at λ = 407 nm of (SCN)_2_^•–^. *A*[(SCN)_2_^•–^]_X_ represents the absorption maximum at λ = 561 nm or, respectively, at λ = 407 nm of (SCN)_2_^•–^ in the presence of an organic reactant in the solution, which decreases by increasing the reactant concentration. *k*_R-1_ represents the second-order rate constant of the organic reactant (R-1). *k*_REF_ denotes the reference rate constant for the reaction (R-2). All calculations were based on the temperature-dependent reference rate constant (eq 2) introduced by Zhu et al.^16^
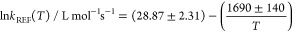
2

A linear relationship with a slope of *k*_R-1_/*k*_REF_ results from the ratios of the absorption maxima (*A*[(SCN)_2_^•–^]_0_/*A*[(SCN)_2_^•–^]_X_) of each solution to the corresponding concentration ratio of organic and reference compounds, [RH]/[SCN^–^]. All reported rate constant errors are statistical errors using Student’s t-distribution with 95% confidence interval. The aqueous solutions were freshly prepared at the respective pH value and measured within 15 min to avoid possible side reactions.

The experiment with lactic acid as the organic compound was carried out at an observation wavelength of 407 nm. The experiment with the other two compounds was carried out at an observation wavelength of 560 nm.

## DFT Calculations

### Density Functional Theory (DFT) Calculation

The DFT calculations were conducted in the present study to determine the energy barrier of the ^•^OH radical induced H atom abstraction reaction using the Gaussian 16 package.^17^ The standard 6-311++g(d,p) basis set in combination with the M06-2X functional was applied to optimize the geometry and calculate the vibration frequency for all reactants, intermediates, and products.^18^ In order to obtain more precise energies, the single-point energies (SPEs) were determined with the M06-2*X*/6-311++g(3df,2p) set. The intrinsic reaction coordinate (IRC) calculations were analyzed to allow verification of the transition state associated with the corresponding reactants and products.^19^ A better description of the solvent effect was obtained by using the continuous solvation model (SMD) within a self-consistent reaction field (SCRF) theory for the calculations.^20,21^ Further details on the DFT calculations has been provided in a previous study.^22−24^ There, the energy barriers (*E*_B_) were calculated as follows:
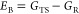
3
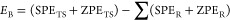
4

*G*_R_ and *G*_TS_ are the free standard Gibbs energies, SPE_R_ and SPR_TS_ are the single-point energies, and ZPE_R_ and ZPE_TS_ are the zero-point energies of the reactants and of the transition state. The energy barrier represents the minimum energy required for a chemical reaction to occur. This can be described as a potential field that controls the transformations of molecules and from which the position of the hydrogen atom can be predicted, which is preferably abstracted by a radical.

## Results and Discussion

### Molecular Composition of Aqueous Solutions and the Mechanism of Oxidation

**Scheme 1:**
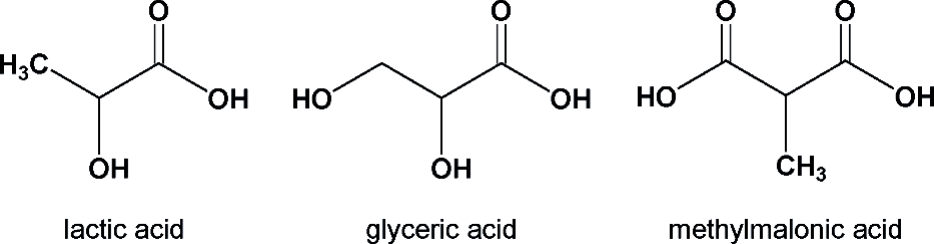
Chemical Structures of the C_3_ Organic Acids Lactic Acid and Glyceric Acid and the C_4_ Carboxylic Acid Methylmalonic Acid Investigated as Reactants within This Study

In aqueous solution, organic acids (Scheme 1) exhibit a well-known acid–base behavior, characterized by the formation of a protonated form and a deprotonated form, depending on the pH value.^6,12^ In general, the ^•^OH radical reacts in aqueous solution mainly with organic carboxylic acid in a H-atom abstraction mechanism.^6,12^

**Scheme 2:**
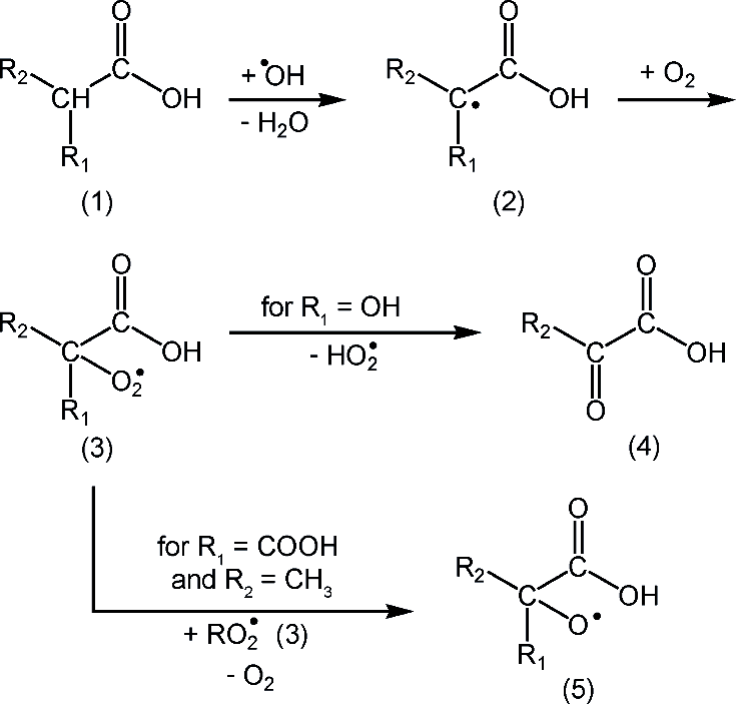
Proposed Oxidation Mechanism of Carboxylic Acids by ^•^OH in the Aqueous Phase

^•^OH attack is most likely to occur at the C–H bond with the lowest bond dissociation energy (BDE), with the BDE following the general order OH > CH_3_ > CH_2_ > CH.^25^ The subsequent species is the alkyl radical (Scheme 2, 2) formed after abstraction of the H atom. In the presence of O_2_ (*c*(O_2_) = 2.6 × 10^–4^ M at 298 K)^26^ the peroxyl radical is formed next.^27^ Depending on the structure, the peroxyl radicals (Scheme 2, 3) can either recombine (Scheme 2, 3) or, if an α-hydroxy peroxyl radical has been formed, decompose into a HO_2_^•^ radical and the corresponding carbonyl-containing molecule (Scheme 2, 4). In the case of lactic acid, one important oxidation product is pyruvic acid. Glyceric acid is most likely to have the ^•^OH radical reacting in the α-position, eventually producing hydroxy-pyruvic acid and HO_2_^•^ radicals. In the case of methylmalonic acid, the oxidation could also lead to pyruvic acid as the preferred pathway might be recombination of the peroxyl radical. Since there is no OH group as a substituent at the α-position in methylmalonic acid, and the substituent R_1_ and R_2_ are not hydrogen atoms, the recombination leads to alkoxyl radicals (Scheme 2, 5). The alkoxyl radical (Scheme 2, 5) can subsequently be expected to decompose by decarboxylation to form pyruvic acid if the COOH group is removed, or if the CH_3_ group is removed, mesoxalic acid would be the reaction product.^28^ In solutions in which organic compounds are present in high concentrations, the alkoxyl radical (Scheme 2, 5) might, possibly, also undergo a bimolecular H-abstraction reactions^28^ in competition to the above unimolecular decompositions.

Since the ^•^OH radicals are generated by the photolysis of H_2_O_2_ at λ = 248 nm, the UV/vis absorption of the organic reactant, in this case the carboxylic acids, could lead to the decrease of the ^•^OH radical concentration, resulting in an overestimation of the measured rate constant due to the internal filter effect.^10^ The method of the competition kinetics is based on the assumption that [^•^OH]_0_ ∼ [*A*(SCN)_2_·^–^]_0_ (eq -1) is constant over the course of the measurement series. If the reactant concentration used in combination with the molar absorption coefficient (ε) is too high, the reduction of ^•^OH radicals is no longer negligible and leads to an increase in the calculated second-order rate constant. It is therefore necessary to characterize the absorption of the target compounds at 248 nm to correct for this effect. To address this potential issue, molar absorption coefficients (ε) of organic compounds were measured across a wavelength range of 200 to 400 nm (Supporting Information). All carboxylic acids studied in this work exhibited pH-dependent ε values (λ = 248 nm) as follows: Lactic acid ε(pH 1.5) = 0.6 L mol^–1^ cm^–1^ and ε(pH 9) = 0.4 L mol^–1^ cm^–1^; glyceric acid ε(pH 1) = 0.7 L mol^–1^ cm^–1^ and ε(pH 8) = 2.4 L mol^–1^ cm^–1^; methyl malonic acid ε(pH 1) = 0.6 L mol^–1^ cm^–1^ and ε(pH 4.4) = 0.9 L mol^–1^ cm^–1^ and ε(pH 8.5) = 2.4 L mol^–1^ cm^–1^. These values suggest that the coabsorption effect can be considered negligible based on the concentrations applied in the measurements.

## DFT Calculations

DFT calculations were used to calculate the effect of protonation and deprotonation of lactic acid on the ^•^OH radical reaction in the aqueous phase (Table 1).

**Table 1:** Calculated Energy Barrier in kJ mol^–1^ of the ^•^OH Radical Induced the H Atom Abstraction Reaction in the Aqueous Phase

reactant	group	protonated form	monoanionic form	dianionic form
lactic acid	-COOH	68.7		
	–OH	58.5	46.6	
	-CH_3_	44.7	34.7	
	-CH	35.5	23.4	
glyceric acid	-COOH	50.2		
	-CH	33.5	30.0	
	–OH	60.9	55.4	
	-CH_2_	30.5	19.7	
	–OH	50.9	39.0	
methylmalonic acid	-COOH	69.3		
	-CH	45.9	33.9	26.0
	-CH_3_	43.1	43.4	30.2
	-COOH	70.2	53.2	

The calculated energy barriers are discussed and related to the effects of protonation and deprotonation in the individual organic compound sections.

### T and pH Dependency of the ^•^OH-Initiated Reactions in the Aqueous Phase

#### Lactic Acid

**Figure 1:**
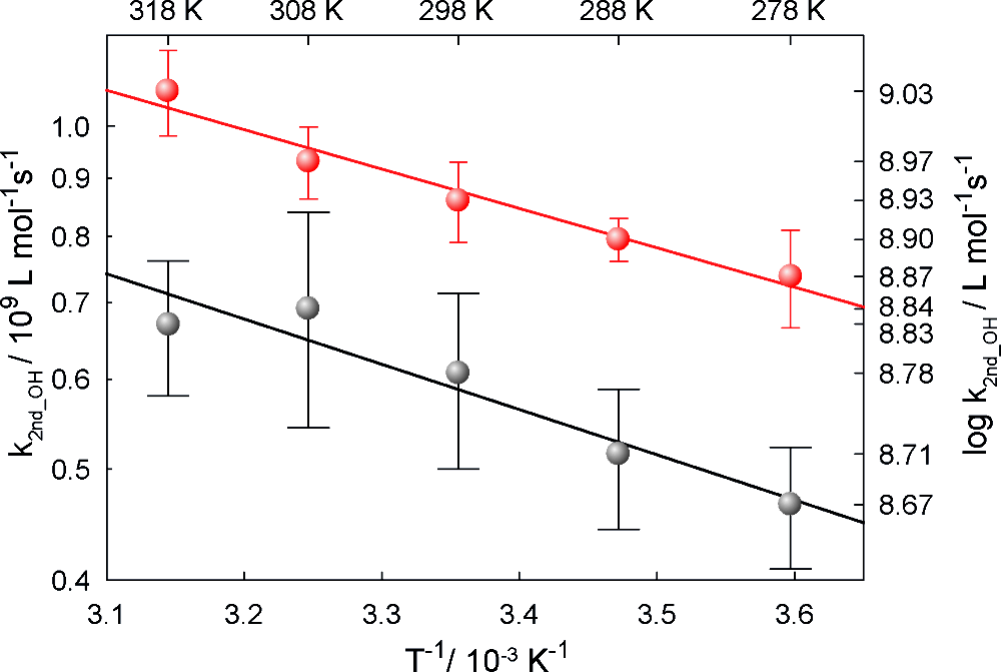
Determined second-order rate constants for ^•^OH reactions with lactic acid at pH 1.5 (black) and lactate at pH 9 (red) in a temperature range of 278 ≤ *T* ≤ 318 K.

The observed *T*-dependent ^•^OH rate constants (*k*_second_) with lactic acid and lactate are shown in Figure 1 as well as in Table S1. Lactic acid owns two p*K*_a_ values 3.86 and 15.1.^29^ The lower p*K*_a_ value refers to the protonation and deprotonation of the carboxylic acid group forming lactate, while the higher p*K*_a_ value refers to the hydroxyl group (Figure S2, Supporting Information). The obtained rate constants at *T* = 298 K can be given *k*(pH 1.5) = (6.1 ± 1.1) × 10^8^ L mol^–1^ s^–1^ for the protonated carboxylic acid group and *k*(pH 9) = (8.6 ± 0.7) × 10^8^ L mol^–1^ s^–1^ for lactate.

The following Arrhenius equations (eq 5) and (eq 6) describe the temperature-dependent change of rate constant.
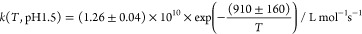
5
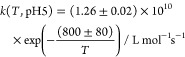
6

In the case of fully protonated lactic acid, the following average Gibbs energy barriers were determined by DFT calculations for the H atom abstraction reaction (Table 1). The lowest energy was calculated at the C–H bond in the α position with 35.5 kJ mol^–1^, followed by the C–H bond of the methyl group with 44.7 kJ mol^–1^, followed by the O–H bond at the hydroxyl group with 58.5 kJ mol^–1^ and finally by the O–H bond of the carboxylic acid with an energy value of 68.7 kJ mol^–1^. Compared to the lactate, which exhibits the following energies: 23.4 kJ mol^–1^ at the C–H bond in the α position, followed by 34.7 kJ mol^–1^ at the C–H bond of the methyl group and followed by 46.6 kJ mol^–1^ at the O–H bond at the hydroxyl group. The deprotonation of the carboxylic acid leads to an increase in the experimentally observed rate constant by 29%, which can be explained by the electronic effect of the carboxylate group and the reduction in the Gibbs energy of the CH group by 34%, the CH_3_ group by 23%, and the OH group by 20%.

#### Glyceric Acid

In the case of glyceric acid, the ^•^OH radical rate constants were also measured as a function of *T* and pH to investigate the change in reactivity due to deprotonation of the carboxylic acid group. Glyceric acid has a p*K*_a_ value of 3.52,^30^ which refers to the protonation and deprotonation of the carboxylic acid group (Supporting Information). Under strong alkaline conditions, the hydroxyl groups are also deprotonated, which is similar to the behavior of lactic acid. For the protonated glyceric acid, the rate constant was measured to be *k*(pH 1) = (1.4 ± 0.1) × 10^9^ L mol^–1^ s^–1^, while for the deprotonated form, the rate constant was obtained to be *k*(pH 8) = (2.4 ± 0.4) × 10^9^ L mol^–1^ s^–1^.

**Figure 2:**
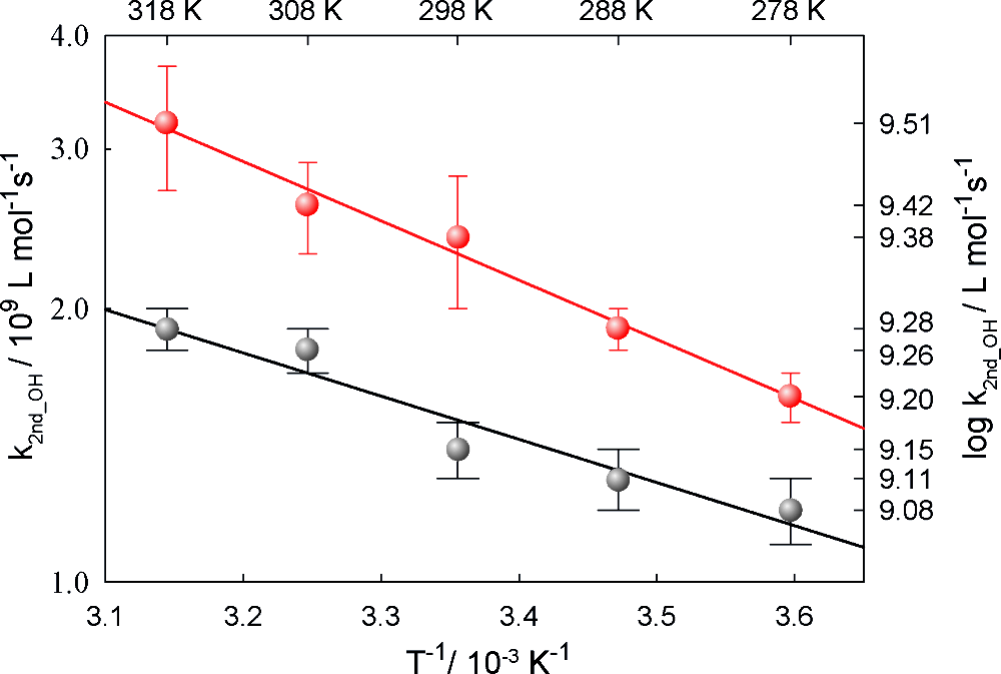
Arrhenius plot of the determined second-order rate constants for ^•^OH reactions with glyceric acid at pH 1 (black) and glycerate at pH 8 (red).

In Figure 2 and in Table S1 the *T*- and pH-dependent behaviors of the obtained rate constants are depicted.

The following Arrhenius equations of (eq 7) the protonated and (eq 8) the deprotonated form, respectively, were derived:
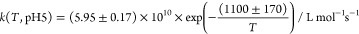
7
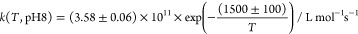
8From the DFT calculations we have the following results (Table 1) for the protonated form: 30.5 kJ mol^–1^ at the CH_2_ group in β-position, 33.5 kJ mol^–1^ at the CH group in the α-position, followed by 50.2 kJ mol^–1^ at the carboxyl group, followed by 50.9 kJ mol^–1^ at the O–H bond in β-position and 60.9 kJ mol^–1^ at the O–H bond in α-position. In the case of the deprotonated glycerate, the following energies were calculated: 19.7 kJ mol^–1^ at the CH_2_ group in the β-position, 30.0 kJ mol^–1^ at the CH group in the α-position, followed by 39.0 kJ mol^–1^ at the O–H bond in the β-position and followed by 55.4 kJ mol^–1^ at the O–H bond in the α-position.

The deprotonation of the glyceric acid also leads to an increase in the rate constant by 42%. The reduction in the Gibbs energy at the most favorable position of the H atom abstraction can be given as follows: CH_2_ group by 35%, CH group by 10%, β–OH group by 23%, and α–OH group by 9%.

#### Methylmalonic Acid

Methylmalonic acid as a dicarboxylic acid has two p*K*_a_ values^30^ of 3.07 and 5.76, so the second-order rate constants were measured at 298 K and at the following pH values 1, 4.4, and 8.5, respectively. The following values were obtained *k*(pH 1) = (1.6 ± 0.1) × 10^8^ L mol^–1^ s^–1^, *k*(pH 4.4) = (2.6 ± 0.1) × 10^8^ L mol^–1^ s^–1^, and *k*(pH 8.5) = (6.2 ± 0.4) × 10^8^ L mol^–1^ s^–1^ (Supporting Information). At pH 4.4, the monoanionic form (AH^–^) is present at 88.1%, at 3.1% in its fully protonated form (AH_2_), and at 8.2% in its fully deprotonated form (A^2–^). The rate constant of the monoanionic form was calculated by using a simple ratio equation (eq 9).
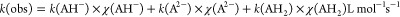
9

**Figure 3:**
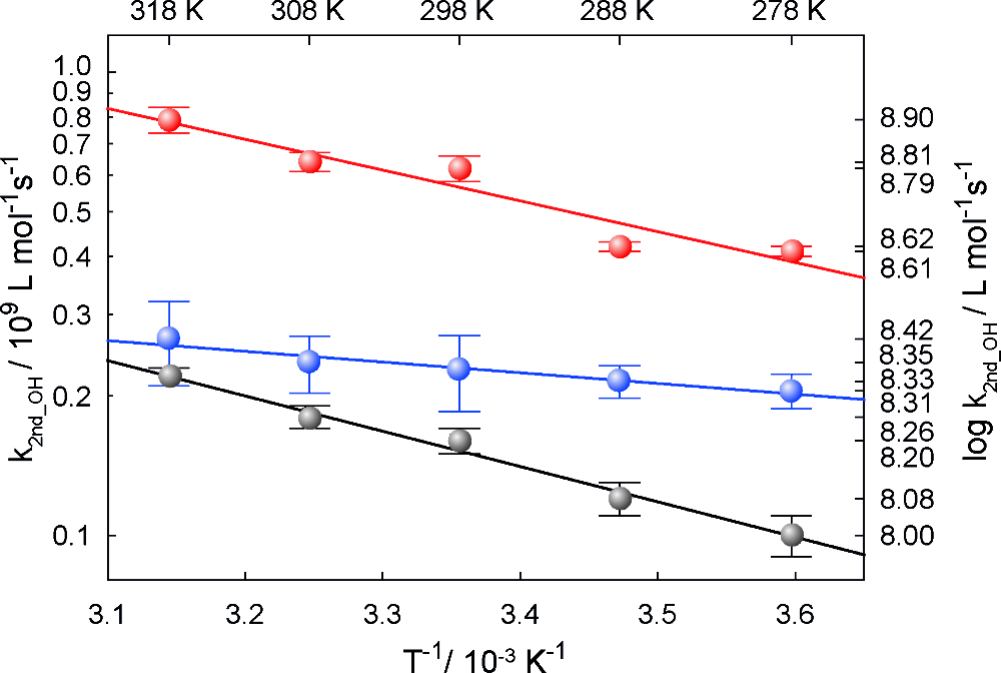
*T*-dependent behavior of second-order rate constants for ^•^OH reactions with the fully protonated form (pH 1) (black), the fully deprotonated form (pH 8.5) (red), and the corrected monoanionic form (blue) of methylmalonic acid.


The following calculated result was obtained *k*(AH^–^) = (2.3 ± 0.4) × 10^8^ L mol^–1^ s^–1^ at *T* = 298 K. The *T*-dependent behavior of the rate constants of ^•^OH radicals with methylmalonic acid is shown in Figure 3 and in Table S1.

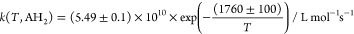
10
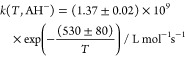
11
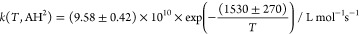
12

The calculated Gibbs energies using the DFT method were determined as follows for the fully protonated methylmalonic acid: 43.1 kJ mol^–1^ at the CH_3_ group, 45.9 kJ mol^–1^ at the CH group, followed by 69.3 kJ mol^–1^ at the first carboxyl group and followed by 70.2 kJ mol^–1^ at the second carboxyl group. Deprotonation to the monoanionic form leads to a decrease in the energies with 33.9 kJ mol^–1^ at the CH group, 43.4 kJ mol^–1^ at the CH_3_ group and followed by 53.2 kJ mol^–1^ at the second carboxyl group. The fully deprotonated form gives a value of 26.0 kJ mol^–1^ for the CH group and a value of 30.2 kJ mol^–1^ for the CH_3_ group. The reduction in the Gibbs energy was calculated as follows: For the CH group 26% for AH^–^ and 43% for A^2–^ and at the CH_3_ group −1% for AH^–^ and 30% for A^2–^. This is compared to increases in the rate constant of 63% for the monoanionic form and 74% for the fully deprotonated form relative to the protonated form.

#### Diffusion-Limiting Rate Constant

Diffusion is known to be a potential factor influencing the reaction rate constants in the aqueous phase. The *T*-dependent diffusion rate constants (*k*_diff_) were calculated by using the Smoluchowski equation. As shown in the Supporting Information, the diffusion rate constants were estimated in the range of 10^9^ – 10^10^ L mol^–1^ s^–1^. The observed rate constants for studied carboxylic acids (lactic acid, glyceric acid, and methylmalonic acid) are at least 1–2 orders of magnitude smaller than *k*_diff_. In conclusion, the oxidation process of these carboxylic acids with ^•^OH in the aqueous phase is not limited by diffusion.

#### Structure–Activity Relationship (SAR) Methods

**Figure 4:**
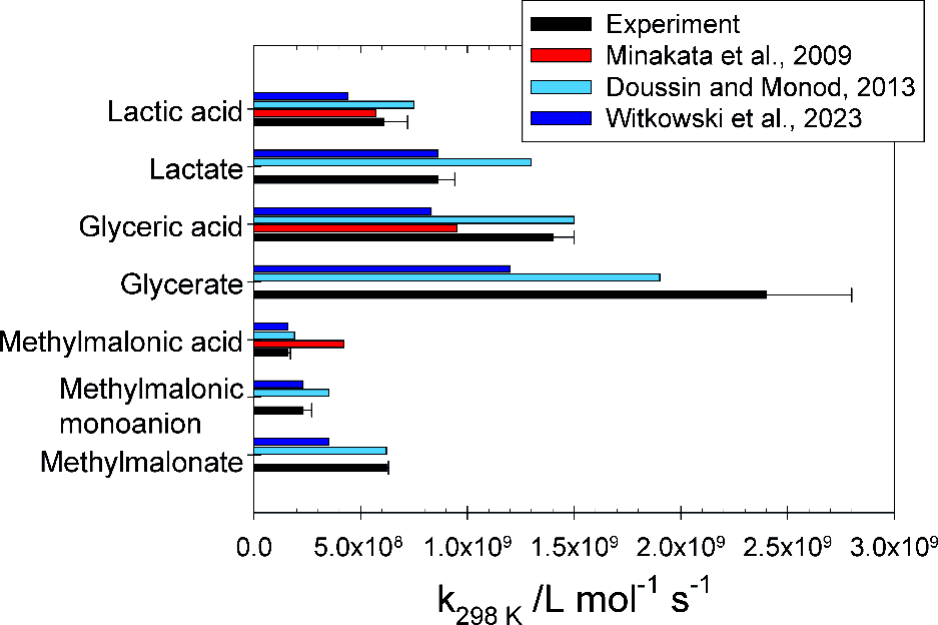
^•^OH radical experimentally determined the rate constants of the present study compared with those calculated using the SAR method (expressed in L mol^–1^ s^–1^).

The structure–activity relationships (SARs) of Minakata et al.,^31^ Doussin and Monod,^32^ and Witkowski et al.^33^ were used to estimate the ^•^OH radical rate constants (Supporting Information). Witkowski et al. updated the contribution factors and the partial reactivity rates from Doussin and Monod using C_2_–C_10_ linear and terpenoid alcohols and diols as an organic reactant with ^•^OH radicals in the aqueous phase. Experimentally determined rate constants in this work are summarized in Figure 4 and Table S3 together with the rate constants predicted by the SAR methods.

Compared to the experimental values, the method of Minakata et al.^31^ gives lower rate constants for lactic acid and glyceric acid, and the pH dependence is not represented in this method. The calculated rate constant for methylmalonic acid is overestimated by a factor of 2.6. The SAR method of Doussin and Monod^32^ shows higher rate constants were determined for lactic acid, lactate, glyceric acid, and glycerate. In the case of methylmalonic acid, the protonated and monoanionic forms are slightly overestimated, while the dianionic form is well-estimated. The optimum level (±20%) of the predicted rate constants for the method developed by Doussin and Monod and further developed by González-Sánchez et al.,^34^ which was later updated by Witkowski et al., is also achieved.^32,33^ Applying the updated rate increments and neighboring factors from Witkowski et al. results in a different pattern in the prediction. The predicted rate constants are lower compared to the experimental values of lactic acid, glyceric acid, and the dianion of methylmalonic acid. However, the values for lactate and the protonated and monoanionic forms of methylmalonic acid are very well-estimated. The deviations of the experimentally determined rate constants from the calculated values reflect the precision level of the SAR method used and its predictive ability, which corresponds to a factor of 0.5 to 2 for the method developed by Minakata.^31^ The deviations from the predicted rate constant by the method of Minakata et al. are within this precision factor.

#### Thermochemical Parameters

The thermochemical parameters are determined using temperature-dependent rate constants *k*(*T*) and the Arrhenius equation (eq 13).
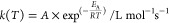
13

*E*_A_ represents the activation energy, *R* the gas constant according to the ideal law, *T* the temperature, and *A* the pre-exponential factor.^35^ The equations for the calculation of the activation enthalpy (Δ*H*^⧧^), the activation entropy (Δ*S*^⧧^), and the Gibbs free energy of activation (Δ*G*^⧧^) are as follows:
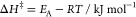
14
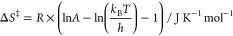
15
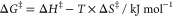
16*k*_B_ is the Boltzmann constant (*k*_B_ = 1.38 × 10^23^ J K^–1^), and *h* is the Planck constant (*h* = 6.626 × 10^–34^ J s). The obtained activation parameters for the reactions investigated are summarized in Table 2.

**Table 2:** Overview of Experimentally Derived Activation Parameters of the ^•^OH Radical Reactions in the Aqueous Phase from This Study

reactants	*E*_A_/kJ mol^–1^	*A*/L mol^–1^ s^–1^	Δ*H*^⧧^/kJ mol^–1^	Δ*S*^⧧^/J K^–1^ mol^–1^	Δ*G*^⧧^/kJ mol^–1^
lactic acid	8 ± 1	(1.3 ± 0.1) × 10^10^	5 ± 1	–(60 ± 2)	23 ± 6
lactate	7 ± 1	(1.3 ± 0.1) × 10^10^	4 ± 1	–(60 ± 1)	22 ± 3
glyceric acid	9 ± 1	(6.0 ± 0.2) × 10^10^	7 ± 1	–(47 ± 1)	21 ± 5
glycerate	13 ± 1	(3.6 ± 0.1) × 10^11^	10 ± 1	–(32 ± 1)	20 ± 2
methylmalonic acid	15 ± 1	(5.5 ± 0.1) × 10^10^	12 ± 1	–(48 ± 1)	26 ± 2
methylmalonic monoanion	4 ± 1	(1.4 ± 0.1) × 10^9^	2 ± 1	–(78 ± 1)	25 ± 5
methylmalonic dianion	13 ± 1	(9.6 ± 0.4) × 10^10^	10 ± 2	–(43 ± 2)	23 ± 6

*E*_A_ is the energy barrier that a reaction must overcome when the reaction proceeds from reactants to products. Values between 4 and 12 kJ mol^–1^ were derived from the results of this study. The Arrhenius equation (eq 12) shows that the rate constants are directly proportional to *A* and can be described as an empirical constant if each collision leads to a reaction. The obtained values of *A* for all radical-initiated reactions ranged from 10^9^ to 10^11^ L mol^–1^ s^–1^, which is comparable to those of similar radical reactions.^15,36,37^ The activation enthalpy (Δ*H*^⧧^) can be calculated from (eq 14) and represents the change in enthalpy of the activated complex. It can also be considered as the degree of stability of the activated complex. According to (eq 15), the entropy of activation (Δ*S*^⧧^) can be calculated and characterizes a certain degree of randomness or order in the transition of the reactants from the ground state to the transition state (TS).

All Δ*S*^⧧^ exhibit negative values (Table 2), indicating a more ordered TS compared with the reactants. The Gibbs activation energy (Δ*G*^⧧^) of the oxidation reactions listed in Table 2 has positive Δ*G*^⧧^ values, which indicates the structural and energetic similarity of the activated complex and the products formed. The experimentally determined Δ*G*^⧧^ varies only slightly within the determined error limits from 20 to 26 kJ mol^–1^ and is comparable with other ^•^OH radical reactions.^7,15^

#### Comparison with Reported Literature Values

**Figure 5:**
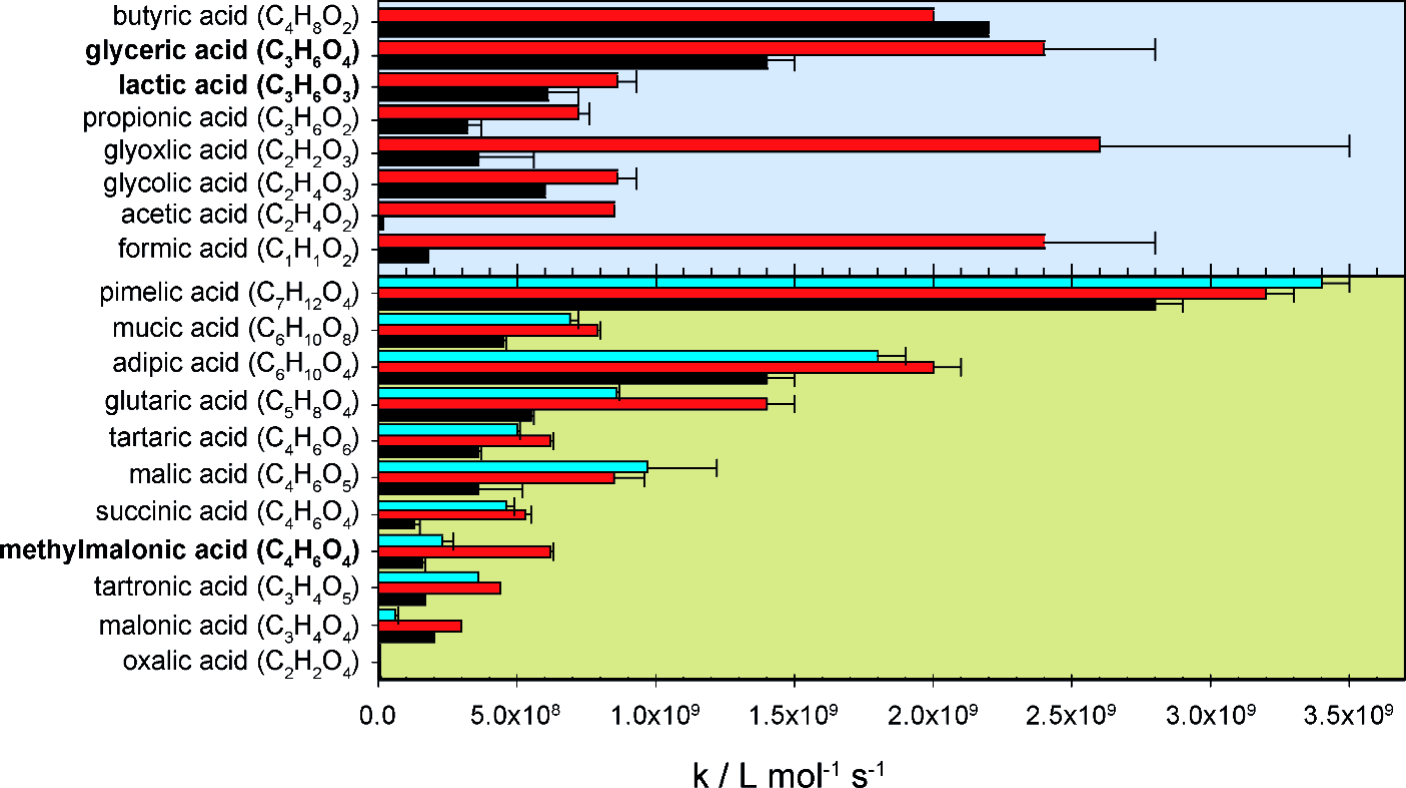
^•^OH radical experimentally determined rate constants of the present study (in bold) compared with literature values of monocarboxylic acids (MCAs) and dicarboxylic acids (DCAs) (Table S4). Black (■) is the protonated form, red (■) is the fully deprotonated form of MCA and DCA, and blue (■) is the monoanionic form of the DCA.

Figure 5 and Table S4 summarize the second-order rate constants of the reactions of the ^•^OH radical with the aforementioned compounds and additionally similar reactants. As the values of the rate constants in numerous publications show, the ^•^OH radical rate constants are in the range of 10^7^ to 10^9^ L mol^–1^ s^–1^.^6,7,15,38,14^ For the compounds shown, the rate constant for reactants without further substituents increases with the number of carbon atoms, as is the case with simple, unsubstituted alkanes and alcohols.^38^ A deviation from this pattern can be observed in the branched and substituted MCA and DCA. The reason for this deviation can be explained by the fact that more complex molecules with larger branching and different substituents, such as −CH_3_, −OH, −COOH, or −COO^–^ groups, exert either activating or deactivating effects on specific hydrogen atom positions in the molecule, leading to the observed nonlinear behavior.

However, a general trend can be described: When the carboxylic acid function is deprotonated, the rate constants increase due to the change in the electronic effect of the carboxyl group combined with the possibility of a direct single electron transfer taking place. Formic acid, acetic acid, and glyoxylic acid exhibit a very strong increase of the rate constants (Table S4) due to the deprotonation with a ratio of A^–^/HA of 17.8, 53.1, and 7.2, respectively.^38,39^ The deprotonation of glycolic acid, propionic acid, lactic acid, and glyceric acid shows a less strong effect on the rate constants, with a ratio A^–^/HA of 1.4, 2.3, 1.4, and 1.7. A comparison with the rate constants for lactic acid determined by Martin et al. shows very good agreement with the values measured in this study (Table S4) and a similar ratio of 1.5.^40^ Also the rate constant of glycolic acid is comparable to the obtained result of lactic acid as the substitution of a hydrogen atom by a CH_3_- group has no significant impact. Propionic acid as a basic structure shows a smaller rate constant compared to lactic acid and glyceric acid, since the influencing effect of the OH- substituents is not present. The difference of butyric acid from the general trend could be explained by the use of two different methods and competition kinetics reference reactants.^38^ In the case of the DCA shown in Figure 5 and in Table S4 a similar trend can be observed. The rate constant increases with the length of the carbon chain and is influenced by the electronic effects of the substituents, if present. Oxalic acid as the smallest DCA (C_2_) has a rate constant of *k* = (4.6 ± 1.5) × 10^7^ L mol^–1^ s^–1^, while the deprotonated form reacts 42 times slower. However, this behavior is only observed in the ^•^OH radical reaction of oxalic acid; otherwise, the deprotonated form reacts faster than the fully protonated form and the anion form as discussed for DCA. In the special case of oxalate and oxalic acid, the ^•^OH radical must undergo an electron transfer reaction with the deprotonated form of the carboxyl group. As soon as another C atom is introduced into the carbon chain of the DCA, the main reaction mechanism probably changes to H atom abstraction with a remaining smaller contribution of electron transfer. For the H-abstractions, the electronic effect of the protonation/deprotonation of the carboxyl group influences the bond dissociation energy of the most likely abstractable C–H position in the molecule leading to the increase of the rate constant of the fully deprotonated form. The rate constants of the monoanionic form of DCA are often lower than those of the fully deprotonated form. Malic acid and pimelic acid show a different behavior. The anionic form reacts somewhat faster, but the error ranges of the two values overlap for malic acid and pimelic acid, so that the behavior of these DCAs is not clear.

### Atmospheric Implications

The kinetic measurements conducted in this study allow for the estimation of the lifetime (τ) of the investigated carboxylic acids in the atmospheric aqueous phase, i.e., liquid aerosols and clouds (Table 3). The lifetimes were determined by utilizing the inverse relationship between the obtained second-order rate constant and atmospheric concentration of the ^•^OH radical. The ^•^OH radical concentrations were simulated with CAPRAM 4.0 to describe the atmospheric chemistry in the aqueous phase in combination with the Master Chemical Mechanism version 3.3.1 (MCMv3.3.1) to describe the chemistry in the gas phase.^9,41^

**Table 3:** Atmospheric Lifetimes in the Aqueous Phase for Model Conditions: Aerosols and Clouds in Urban and Remote Areas Condition and Measured ^•^OH Radical Concentration at *T* = 288 K and pH Values^13,44^

conditions	urban cloud	remote cloud	urban aerosol	remote aerosol	measured OH conc./mol L^–1^ ^44^
Lactic acid					
pH 5	2.25 years	1.2 months	14.8 months	11.3 days	5.9 months
pH 1	**/**	**/**	22.3 months	16.9 days	8.9 months
Glyceric acid					
pH 5	11.2 months	14.8 days	6.2 months	4.7 days	3.6 months
pH 1	**/**	**/**	8.9 months	6.8 days	2.5 months
Methylmalonic acid					
pH 5	6.4 years	3.4 months	3.5 years	1.1 months	3.2 years
pH 1	**/**	**/**	8 years	2.4 months	1.4 years

For the different scenarios, the following average ^•^OH radical model concentrations were used: 1.1 × 10^–15^ mol L^–1^ for an urban cloud, 2.5 × 10^–14^ mol L^–1^ for a remote cloud, 2.0 × 10^–15^ mol L^–1^ for an urban aerosol, 7.9 × 10^–14^ mol L^–1^ for a remote aerosol.^13^

Compared with concentrations measured in cloud droplets and aerosols, the values above are in good agreement ((0.5 to 7.7) × 10^–15^ mol L^–1^).^42−44^ Assuming that the ^•^OH radicals present react exclusively with the dicarboxylic acid, the lifetimes in the aqueous phase were determined using the rate constants at *T* = 288 K to illustrate the atmospheric average temperature for the lower troposphere.

In urban areas, the typical pH value of clouds ranges from 4 to 6,^6,7^ where the oxidation of carboxylic acids is primarily dominated by their carboxylate forms. Thus, the rate constants for *T* = 288 K and pH 5 were calculated with the speciation given in the Supporting Information. Under urban cloud conditions, the ^•^OH radicals react with lactic acid, glyceric acid, and methylmalonic acid, potentially extending their atmospheric lifetime from months to years. In remote areas, the average concentration of free radicals increases due to a smaller efficiency of all the combined ^•^OH sink reactions compared to that in the urban case. As a consequence, the lifetime of the acids investigated here under remote cloud conditions increases to days or months.

Compared to clouds, the pH in aerosols decreases drastically to the acidic range of the pH scale; thus, carboxylic acids are expected to be present in their protonated form within the aerosol, leading to a slight decrease in the rate constant. However, as the pH value of the aerosol covers a wide range from 0 ≤ pH ≤ 5, the lifetime was calculated for pH 5.^45,46^ The radical concentration in aerosols is expected to be higher, resulting in shorter lifetimes of months in urban aerosols and days under remote aerosol conditions.

In a more recent study, an ^•^OH radical concentration of 5 × 10^–15^ mol L^–1^ was measured in aerosol particles^44^ and used here for the calculation of the lifetime (Table 3).

However, using the liquid water content (LWC) range of 1 × 10^–5^ to 1 g m^–3^ from aerosol and cloud measurements^6,7^ and the estimated Henry’s law constants of the investigated carboxylic acids,^8^ it is shown that lactic acid, glyceric acid, and methylmalonic acid are present in the cloud phase. Since the solubility of lactic acid and glyceric acid is too small compared to methylmalonic acid using the LWC 1 × 10^–5^ g m^–3^, only methylmalonic acid remains in the aerosol, while the other two carboxylic acids evaporate into the gas phase as the cloud evaporates.

## Conclusion

Rate constants for the reactions of the ^•^OH radical with lactic acid, glyceric acid, and methyl malonic acid in aqueous solution were determined using an LFP-LPA setup combined with the thiocyanate competition kinetics method. The temperature-dependent second-order rate constants were measured from 278 to 318 K, leading to the derivation of Arrhenius expressions that describe the temperature dependence of the reaction rate constants. The observed rate constants of the carboxylic acids exhibit typical values for radical-driven H atom abstraction reactions, with *k*_second_ for ^•^OH radicals ranging from 10^8^ to 10^9^ L mol^–1^ s^–1^, and will enable the experiment-based implementation of the kinetics of these reactions in respective atmospheric multiphase models. The rate constants are highest for the deprotonated form, *k*(A^2–^), followed by the half-protonated form, *k*(HA^–^), and the fully protonated form, *k*(H_2_A). The energy barriers of the ^•^OH radical reaction were calculated using density functional theory (DFT) showing a significant change for the different molecular groups for the protonated and deprotonated carboxylic acids. The kinetic data predicted from the structure–activity relationships agree well with the measured ^•^OH radical rate constants in terms of the predictive ability and degree of precision. Atmospheric lifetimes of lactic acid, glyceric acid, and methylmalonic acid in the aqueous and aerosol phases were calculated at *T* = 288 K for different atmospheric scenarios to provide a more accurate description of the losses of the studied carboxylic acids due to oxidation by ^•^OH radicals.

## Supporting Information Available

Acid–base equilibria of the carboxylic acids, UV/vis spectra, calculation of diffusion rate constants and Structure–Activity Relationship (SAR) for estimating the rate constants of ·OH radicals (PDF)
