# Supplementary material for: T- and pH-Dependent Hydroxyl-Radical Reaction Kinetics of Lactic Acid, Glyceric Acid, and Methylmalonic Acid in the Aqueous Phase
Source: J Phys Chem A. 2025 Feb 14;129(8):1983–92. doi: 10.1021/acs.jpca.4c08063 (PMC11874031; doi:10.1021/acs.jpca.4c08063)
Supplement: Supplementary file 1 — jp4c08063_si_001.pdf [file jp4c08063_si_001.pdf]

Supplementary Information for

# T- and pH- dependent Hydroxyl-Radical Reaction Kinetics of Lactic Acid, Glyceric Acid, and Methylmalonic Acid in the Aqueous Phase

Yuehuan Hu[a, b], Yimu Zhang[b, a], Liang Wen[b, c], Thomas Schaefer[b], and Hartmut Herrmann[a, b*]

[a] School of Environmental Science and Engineering, Shandong University, Qingdao 266237, China

[b] Atmospheric Chemistry Department (ACD), Leibniz-Institute for Tropospheric Research (TROPOS), Permoserstraße 15, 04318 Leipzig, Germany

[c] now at: Chinese Research Academy of Environmental Sciences (CRAES), Beijing 100012, China

* Corresponding author phone: +49 341 2717 7024; fax: +49 341 2717 99 7024; e-mail: herrmann@tropos.de

**TOC**


1. Dithiocyanate radical anion ($(SCN)_2^{\bullet -}$) in the aqueous phase
2. Acid-base equilibria of the carboxylic acids
3. UV/vis absorption spectra of the investigated compounds
4. Observed T-dependent rate constants of the $^{\bullet}$OH radical reaction in the aqueous phase
5. Calculation of diffusion rate constants
6. Structure-Activity Relationship (SAR) for estimating the rate constants of $^{\bullet}$OH radicals
7. Literature overview

### *Dithiocyanate radical anion ($(SCN)_2^{\bullet-}$) in the aqueous phase*

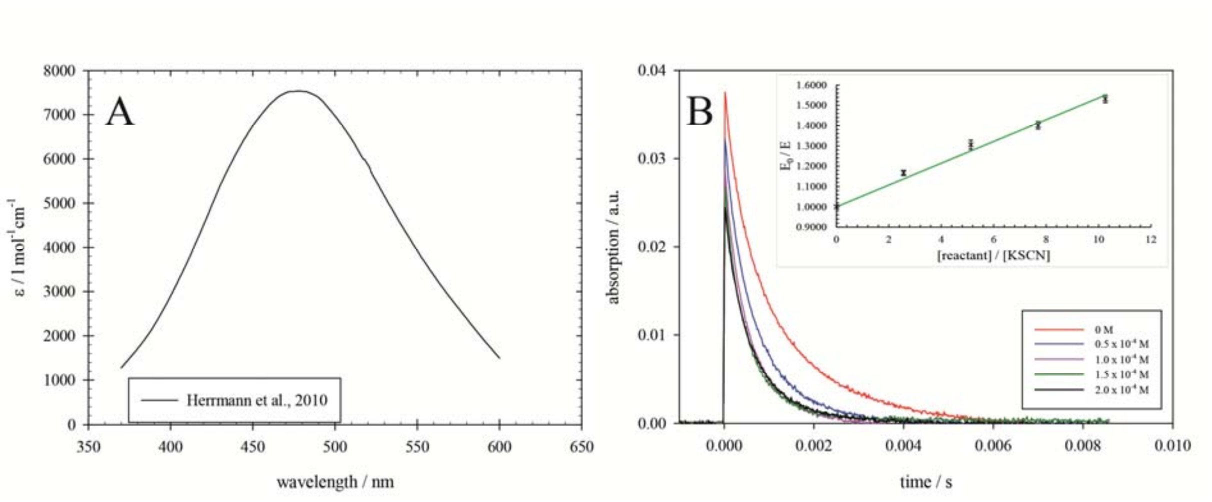


**Figure S1:** A) Averaged absorption spectrum of the dithiocyanate radical anion ($(SCN)_2^{\bullet-}$).[1] B) Absorption-time profiles in the presence of different reactant concentrations using the example of methylmalonic acid at T = 298 K and at pH = 8 and as inset the obtained linear relationship between of the absorption maxima ($A[(SCN)_2^{\cdot-}]_0$/ $A[(SCN)_2^{\cdot-}]_X$) of each solution to the corresponding concentration ratio of organic and reference compounds, $[RH]/[SCN^-]$.

### *Acid-base equilibria of the carboxylic acids*

The acid-base equilibria of the investigated carboxylic acids can be described by the following logarithmic ionization constants (pKa): lactic acid pKa = 3.86 and 15.1, glyceric acid pKa = 3.52 and methylmalonic acid pKa = 2.94 and 5.40.[2, 3]

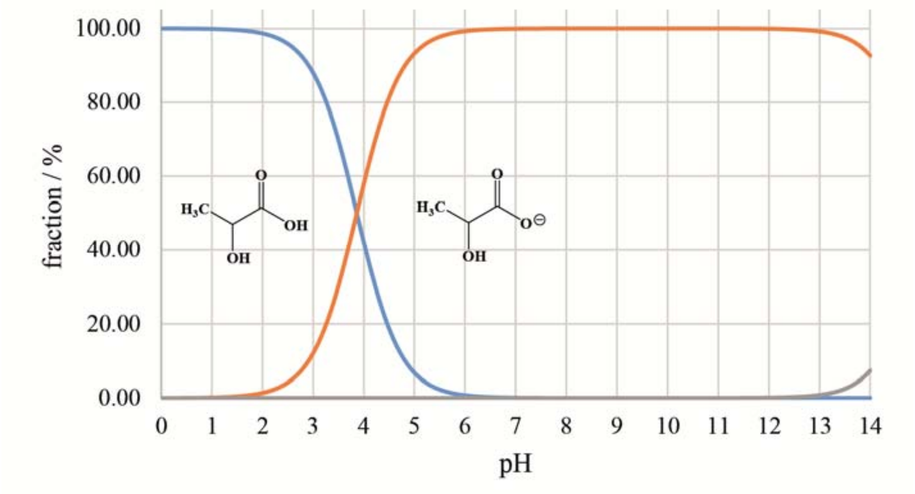


**Figure S2:** Fraction of lactic acid (blue), lactate (orange) and deprotonated lactate (gray) in the aqueous solution, plotted against the pH value.[3]

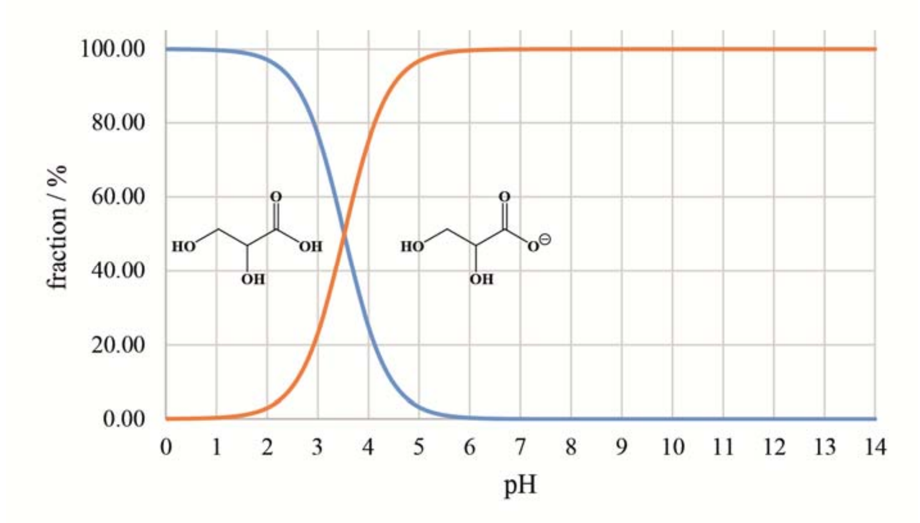


**Figure S3:** Protonated fraction (orange) and deprotonated fraction (blue) of glyceric acid in the aqueous solution, plotted against the pH value.[2]

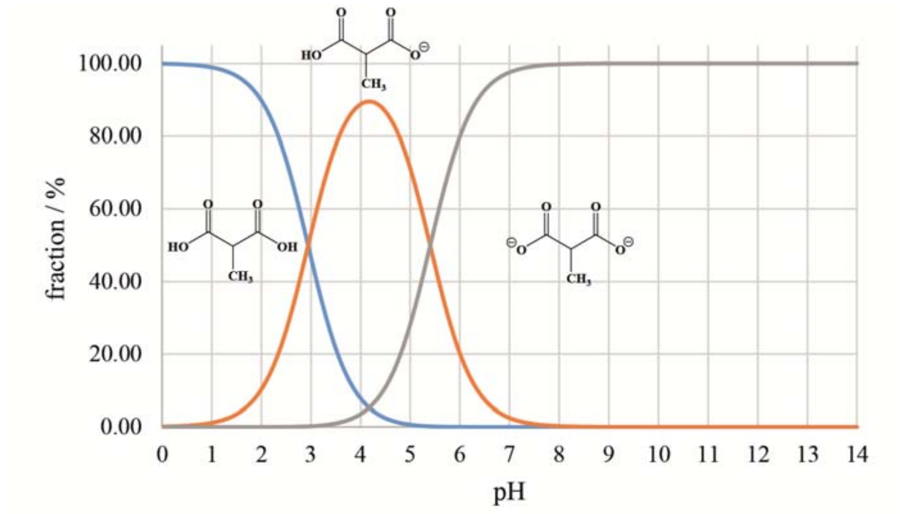


**Figure S4:** Fraction of species $H_2A$ (blue), $HA^-$ (orange) and $A^{2-}$ (gray) of methylmalonic acid in the aqueous solution, expressed in relation to the pH value.[2]

*UV/vis absorption spectra of the investigated compounds*

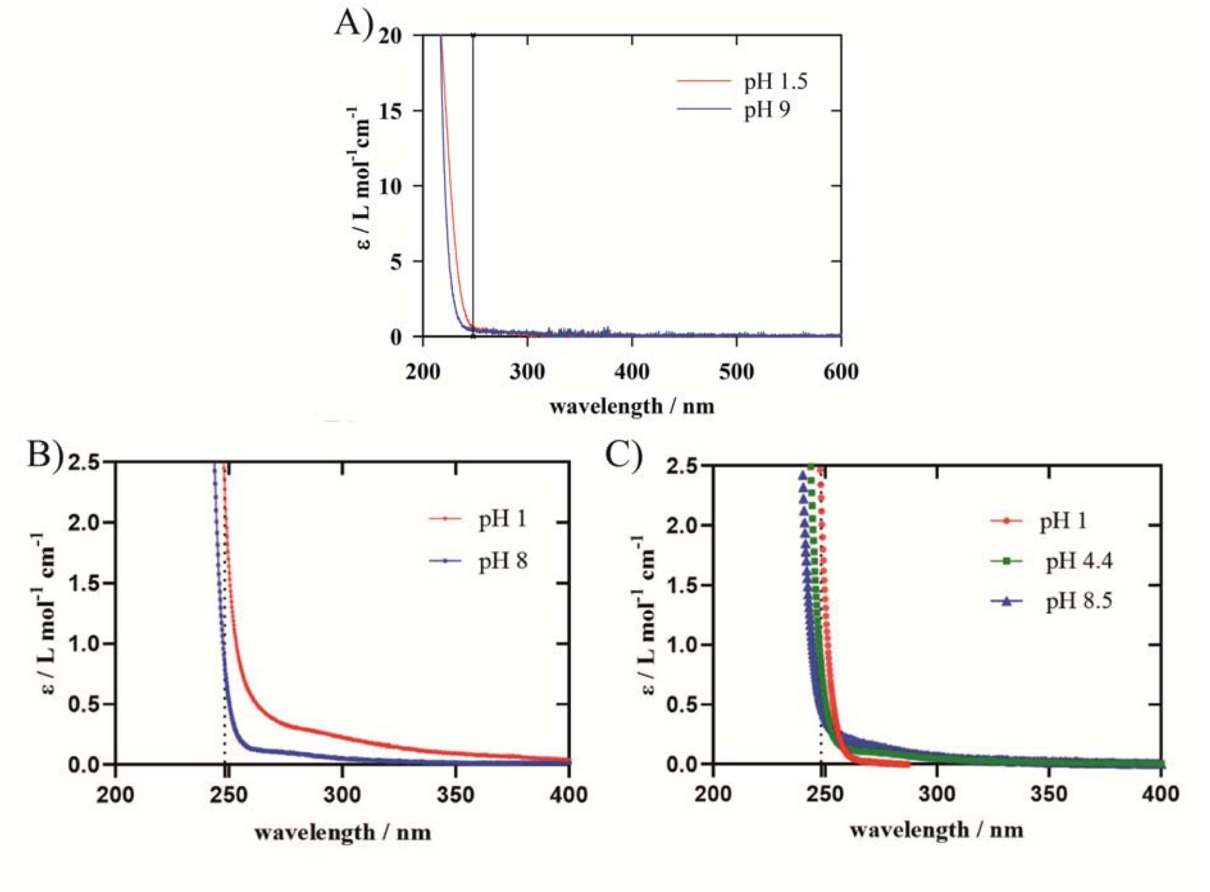


**Figure S5:** UV/vis spectra for (A) lactic acid, (B) glyceric acid and (C) methylmalonic acid.

## ***Observed T-dependent rate constants of the •OH radical reaction in the aqueous phase***

**Table S1:** Observed second-order •OH radical rate constants for the reactions with the investigated carboxylic acids at different temperatures and pH values.

| pH values | $k_{278\ K}$ / L·mol$^{-1}$·s$^{-1}$ | $k_{288\ K}$ / L·mol$^{-1}$·s$^{-1}$ | $k_{298\ K}$ / L·mol$^{-1}$·s$^{-1}$ | $k_{308\ K}$ / L·mol$^{-1}$·s$^{-1}$ | $k_{318\ K}$ / L·mol$^{-1}$·s$^{-1}$ |
|---|---|---|---|---|---|
| | | | **Lactic acid** | | |
| **1.5** | (4.7 ± 0.6) × 10$^8$ | (5.2 ± 0.7) × 10$^8$ | (6.1 ± 1.1) × 10$^8$ | (6.9 ± 1.5) × 10$^8$ | (6.7 ± 0.9) × 10$^8$ |
| **9** | (7.4 ± 0.7) × 10$^8$ | (8.0 ± 0.3) × 10$^8$ | (8.6 ± 0.7) × 10$^8$ | (9.3 ± 0.7) × 10$^8$ | (1.1 ± 0.1) × 10$^9$ |
| | | | **Glyceric acid** | | |
| **1** | (1.2 ± 0.1) × 10$^9$ | (1.3 ± 0.1) × 10$^9$ | (1.4 ± 0.1) × 10$^9$ | (1.8 ± 0.1) × 10$^9$ | (1.9 ± 0.1) × 10$^9$ |
| **8** | (1.6 ± 0.1) × 10$^9$ | (1.9 ± 0.1) × 10$^9$ | (2.4 ± 0.4) × 10$^9$ | (2.6 ± 0.3) × 10$^9$ | (3.2 ± 0.5) × 10$^9$ |
| | | | **Methylmalonic acid** | | |
| **1** | (1.0 ± 0.1) × 10$^8$ | (1.2 ± 0.1) × 10$^8$ | (1.6 ± 0.1) × 10$^8$ | (1.8 ± 0.1) × 10$^8$ | (2.2 ± 0.1) × 10$^8$ |
| **4.4** | (2.1 ± 0.2) × 10$^8$ | (2.2 ± 0.2) × 10$^8$ | (2.3 ± 0.4) × 10$^8$ | (2.4 ± 0.3) × 10$^8$ | (2.7 ± 0.6) × 10$^8$ |
| **8.5** | (4.1 ± 0.1) × 10$^8$ | (4.2 ± 0.1) × 10$^8$ | (6.2 ± 0.4) × 10$^8$ | (6.4 ± 0.3) × 10$^8$ | (7.9 ± 0.5) × 10$^8$ |

## ***Calculation of diffusion rate constants***

The rate constant ($k_{diff}$) of a diffusion-controlled reaction can be estimated using the Smoluchowski's equation.[4]

$$k_{diff} = 4 \times 10^3 \times \pi \times N_A \times (D_{radical} + D_{reactant}) \times (r_{radical} + r_{reactant}) \qquad \text{(Eq. S1)}$$

$N_A$ represents the Avogadro's constant, D represents the diffusion coefficients, r represents radical and reactant radii, and •OH radical radius and the diffusion coefficient are taken from Buxton et al.[5] The modified Stokes-Einstein equation by Wilke and Chang was used to calculate the diffusion coefficients.

$$\mathrm{D} = 7.4 \times 10^{-8} \frac{T\sqrt{X_W M_W}}{\eta_W V_m^{0.6}} \qquad \text{(Eq. S2)}$$

In this case, $X_W$ (dimensionless) is the association parameter and $M_W$ (g mol$^{-1}$) is the molar mass of the solvent. The recommended value of $X_W$ = 2.26 was used for $H_2O$.[6] T (K) is the temperature and $\eta_W$ (10$^{-9}$ kg m$^{-1}$ s$^{-1}$) is the dynamic viscosity of water, which is 1.579, 1.140, 0.890, 0.723, and 0.599 at 278 K, 288 K, 298 K, 308 K, and 318 K, respectively.[7] $V_m$ (m$^3$ mol$^{-1}$) is the molar volume was estimated using the Joback group contribution method, which was estimated using the following relationship, whereas $V_c$ (m$^3$ mol$^{-1}$) is the critical volume.[8]

$$V_m = 0.285 \times V_C^{1.048} \qquad \text{(Eq. S3)}$$

**Table S2:** Overview of molecular properties for estimating diffusion-controlled rate constants.

| molecules | $V_m$ ($cm^3\ mol^{-1}$) | r (nm) | D at 298 K ($m^2\ s^{-1}$) |
|---|---|---|---|
| lactic acid | 89.2[8] | 0.33 | $1.07 \times 10^{-9}$ |
| glyceric acid | 96.6[8] | 0.34 | $1.02 \times 10^{-9}$ |
| methylmalonic acid | 109.5[8] | 0.35 | $0.94 \times 10^{-9}$ |
| $^{\bullet}$OH radical | 26.9[5] | 0.22 | $2.19 \times 10^{-9}$ |

Adopted from Joback method[8] and Buxton et al.[5]

Diffusion rate constants for the $^{\bullet}$OH radical reaction of carboxylic acids at different temperatures were calculated as $k_{diff}(278\ K) = 7.4 \times 10^9\ L\ mol^{-1}\ s^{-1}$, $k_{diff}(288\ K) = 1.0 \times 10^{10}\ L\ mol^{-1}\ s^{-1}$, $k_{diff}(298\ K) = 1.4 \times 10^{10}\ L\ mol^{-1}\ s^{-1}$, $k_{diff}(308\ K) = 1.7 \times 10^{10}\ L\ mol^{-1}\ s^{-1}$, $k_{diff}(318\ K) = 2.2 \times 10^{10}\ L\ mol^{-1}\ s^{-1}$. The results indicate that the measured rate constants are chemically controlled.

***Structure-Activity Relationship (SAR) for estimating the rate constants of $^{\bullet}$OH radicals***

The $^{\bullet}$OH radical-induced oxidation of carboxylic acid by the H-atom abstraction mechanism was estimated at 298 K using Structure-Activity Relationship (SAR). The principle of group additivity applies to organic substances with various functional groups, including aliphatic alkanes, alcohols, organic acids, bases, and polyfunctional compounds. SAR estimation methods proposed by Minakata et al.[9] and Doussin and Monod[10] were used.

The overall rate constant is considered as the sum of the partial reactivity rates, which according to Minakata[9] include the partial reactivity rates of the corresponding H-bearing functional group and the nearest neighbor α-group described by the parameter F (Eq. S4). In addition, Doussin and Monod[10] followed a similar approach, with the difference that they included a second parameter G describing the effect of the nearest neighbor β-group after the directly neighboring α-group (Eq. S5). Moreover, Witkowski et al. recently updated the contribution factors and partial reactivity rates of Doussin and Monod[10].

$$k = \sum k_{2nd,partial} = \sum n_H \times \sum k_{H-baring\ group} \times \sum F\ (\alpha - group) \qquad \text{(Eq-S4)}$$

$$k = \sum k_{2nd,partial} = \sum k_{H-baring\ group} \times \sum F\ (\alpha - group) \times \sum G\ (\beta - group) \qquad \text{(Eq-S5)}$$

**Table S3:** Experimentally determined rate constants of the $^{\bullet}OH$ radical in comparison with those calculated by the SAR method (expressed in L $mol^{-1}$ $s^{-1}$).

| compound | $k_{298\,K,\,\bullet OH}$ experiment | $k_{298\,K,\,\bullet OH}$ Minakata et al.[9] | $k_{298\,K,\,\bullet OH}$ Doussin and Monod[10] | $k_{298\,K,\,\bullet OH}$ Witkowski et al.[11] |
|---|---|---|---|---|
| lactic acid | $(6.1 \pm 1.1) \times 10^8$ | $5.7 \times 10^8$ | $7.5 \times 10^8$ | $4.4 \times 10^8$ |
| lactate | $(8.6 \pm 0.7) \times 10^8$ | | $1.3 \times 10^9$ | $8.6 \times 10^8$ |
| glyceric acid | $(1.4 \pm 0.1) \times 10^9$ | $9.5 \times 10^8$ | $1.5 \times 10^9$ | $8.3 \times 10^8$ |
| glycerate | $(2.4 \pm 0.4) \times 10^9$ | | $1.9 \times 10^9$ | $1.2 \times 10^9$ |
| methylmalonic acid | $(1.6 \pm 0.1) \times 10^8$ | $4.2 \times 10^8$ | $1.9 \times 10^8$ | $1.6 \times 10^8$ |
| methylmalonic monoanion | $(2.3 \pm 0.4) \times 10^8$ | | $3.5 \times 10^8$ | $2.3 \times 10^8$ |
| methylmalonic dianion | $(6.2 \pm 0.4) \times 10^8$ | | $6.2 \times 10^8$ | $3.5 \times 10^8$ |

## *Literature overview*

The following Table S5 gives values for the second order rate constants of the $^{\bullet}OH$ radical reactions with the investigated compounds or structurally similar reactants from the literature.

**Table S4:** Comparison of the determined $k_{2nd}$ with literature data of the $^{\bullet}OH$ radical reactions in aqueous phase at T = 298.15 K.

| reactant | $k_{2nd}$ / L $mol^{-1}$ $s^{-1}$ | remarks | references |
|---|---|---|---|
| | | *monocarboxylic acids (MCA)* | |
| formic acid | $1.8 \times 10^8$ | rec. avg. value<br>T-dep Chin and Wine,[12] | Buxton et al.,[5] |
| formate | $3.2 \times 10^9$ | rec. avg. value<br>T-dep Chin and Wine,[12] | Buxton et al.,[5] |
| | $(2.4 \pm 0.4) \times 10^9$ | LFP, $SCN^-$-C.K., $k_{ref.} = 1.24 \times 10^{10}$ L $mol^{-1}$ $s^{-1}$,<br>pH = 6, A = $(7.9 \pm 0.7) \times 10^{10}$ L $mol^{-1}$ $s^{-1}$,<br>$E_A = 9 \pm 5$ kJ $mol^{-1}$ | Ervens et al.,[13] |
| acetic acid | $1.6 \times 10^7$ | rec. avg. value, at pH = 1 | Buxton et al.,[5] |
| acetate | $8.5 \times 10^8$ | rec. avg. value at pH = nat. | Buxton et al.,[5] |
| glycolic acid | $6.0 \times 10^8$ | rec. avg. value, at pH = 1 - 2.2 | Buxton et al.,[5] |
| glycolate | $(8.6 \pm 0.7) \times 10^8$ | PR, $SCN^-$-C.K., pH = 5.5, $k_{ref.} = 1.1 \times 10^{10}$<br>L $mol^{-1}$ $s^{-1}$ | Bell et al.,[14] |
| | $(8.7 \pm 0.7) \times 10^8$ | FP, pH = 9, 4-FcBA, $k_{ref.} = 1.09 \times 10^{10}$<br>L $mol^{-1}$ $s^{-1}$ | Logan,[15] |
| glyoxylic acid | $(3.6 \pm 0.2) \times 10^8$ | LFP, $SCN^-$-C.K., $k_{ref.} = 1.24 \times 10^{10}$ L $mol^{-1}$ $s^{-1}$,<br>pH = 1, A = $(8.1 \pm 0.4) \times 10^9$ L $mol^{-1}$ $s^{-1}$,<br>$E_A = 8 \pm 3$ kJ $mol^{-1}$ | Ervens et al.,[13] |
| glyoxylate | $(2.6 \pm 0.9) \times 10^9$ | LFP, $SCN^-$-C.K., $k_{ref.} = 1.24 \times 10^{10}$ L $mol^{-1}$ $s^{-1}$,<br>pH = 8, A = $(6.0 \pm 0.4) \times 10^{15}$ L $mol^{-1}$ $s^{-1}$,<br>$E_A = 36 \pm 8$ kJ $mol^{-1}$ | Ervens et al.,[13] |

| reactant | $k_{2nd}$ / L mol$^{-1}$ s$^{-1}$ | remarks | references |
|---|---|---|---|
| propionic acid | (3.2 ± 0.5) × $10^{8}$ | LFP, SCN$^-$-C.K., $k_{ref.}$ = 1.24 × $10^{10}$ L mol$^{-1}$ s$^{-1}$, pH = 1.5, A = (7.6 ± 0.9) × $10^{11}$ L mol$^{-1}$ s$^{-1}$, $E_A$ = 19 ± 8 kJ mol$^{-1}$ | Ervens et al.,[13] |
| propionate | (7.2 ± 0.4) × $10^{8}$ | LFP, SCN$^-$-C.K., $k_{ref.}$ = 1.24 × $10^{10}$ L mol$^{-1}$ s$^{-1}$, pH = 7.0, A = (3.2 ± 0.2) × $10^{11}$ L mol$^{-1}$ s$^{-1}$, $E_A$ = 15 ± 4 kJ mol$^{-1}$ | Ervens et al.,[13] |
| **lactic acid** | **(6.1 ± 1.1) × $10^{8}$** | LFP, SCN$^-$-C.K., $k_{ref.}$ = 1.19 × $10^{10}$ L mol$^{-1}$ s$^{-1}$, pH = 1.5, A = (1.3 ± 0.1) × $10^{10}$ L mol$^{-1}$ s$^{-1}$, $E_A$ = 8 ± 1 kJ mol$^{-1}$ | **This study** |
| | (5.2 ± 0.4) × $10^{8}$ | PR, SCN$^-$-C.K., T = 297.15 K, $k_{ref.}$ = 1.05 × $10^{10}$ L mol$^{-1}$ s$^{-1}$, pH = 1.0, A = (2.3 ± 0.02) × $10^{10}$ L mol$^{-1}$ s$^{-1}$, $E_A$ = 9.3 ± 0.4 kJ mol$^{-1}$ | Martin et al.,[16] |
| | (4.3 ± 0.4) × $10^{8}$ | PR, SCN$^-$-C.K., $k_{ref.}$ = 1.1 × $10^{10}$ L mol$^{-1}$ s$^{-1}$, pH = 1.0 | Adams et al.,[17] |
| **lactate** | **(8.6 ± 0.7) × $10^{8}$** | LFP, SCN$^-$-C.K., $k_{ref.}$ = 1.19 × $10^{10}$ L mol$^{-1}$ s$^{-1}$, pH = 9.0, A = (1.3 ± 0.1) × $10^{10}$ L mol$^{-1}$ s$^{-1}$, $E_A$ = 7 ± 1 kJ mol$^{-1}$ | **This study** |
| | (7.8 ± 0.5) × $10^{8}$ | PR, SCN$^-$-C.K., T = 297.15 K, $k_{ref.}$ = 1.05 × $10^{10}$ L mol$^{-1}$ s$^{-1}$, pH = 6.1, A = (6.1 ± 0.04) × $10^{10}$ L mol$^{-1}$ s$^{-1}$, $E_A$ = 10.8 ± 0.3 kJ mol$^{-1}$ | Martin et al.,[16] |
| | (8.2 ± 0.8) × $10^{8}$ | FP, pH = 9, T = 295.35 K, 4-ferrocenyl-butanoate, $k_{ref.}$ = 1.09 × $10^{10}$ L mol$^{-1}$ s$^{-1}$ | Logan,[15] |
| | 3.0 × $10^{8}$ | PR, ESR-measurement, pH = 11 | Verma and Fessenden,[18] |
| **glyceric acid** | **(1.4 ± 0.1) × $10^{9}$** | LFP, SCN$^-$-C.K., $k_{ref.}$ = 1.19 × $10^{10}$ L mol$^{-1}$ s$^{-1}$, pH = 1.0, A = (6.0 ± 0.2) × $10^{10}$ L mol$^{-1}$ s$^{-1}$, $E_A$ = 9 ± 1 kJ mol$^{-1}$ | **This study** |
| **glycerate** | **(2.4 ± 0.4) × $10^{9}$** | LFP, SCN$^-$-C.K., $k_{ref.}$ = 1.19 × $10^{10}$ L mol$^{-1}$ s$^{-1}$, pH = 8.0, A = (3.6 ± 0.1) × $10^{11}$ L mol$^{-1}$ s$^{-1}$, $E_A$ = 13 ± 1 kJ mol$^{-1}$ | **This study** |
| butyric acid | (2.2 ± 0.2) × $10^{9}$ | γ-R, thymine-C.K., $k_{ref.}$ = 6.4 × $10^{9}$ L mol$^{-1}$ s$^{-1}$, pH = 2 | Buxton et al.,[5] |
| butyrate | (2.0 ± 0.2) × $10^{9}$ | PR, EtOH-C.K., $k_{ref.}$ = 1.9 × $10^{9}$ L mol$^{-1}$ s$^{-1}$, pH = 9 | Buxton et al.,[5] |
| | | *dicarboxylic acids (DCA)* | |
| oxalic acid | (4.6 ± 1.5) × $10^{7}$ | rec. avg. value | IUPAC[19] |
| oxalic acid anion | (5.6 ± 1.8) × $10^{7}$ | rec. avg. value | IUPAC[19] |
| oxalate (di-anion) | (1.1 ±0.4) × $10^{6}$ | rec. avg. value | IUPAC[19] |
| malonic acid | (1.6 ± 1.6) × $10^{7}$ | Fenton reaction, MeOH-C.K., pH = 1.0, $k_{ref.}$ = 9.7 × $10^{8}$ L mol$^{-1}$ s$^{-1}$ | Buxton et al.,[5] |
| | (2.4 ± 1.6) × $10^{7}$ | γ-R, thymine-C.K., $k_{ref.}$ = 6.4 × $10^{9}$ L mol$^{-1}$ s$^{-1}$, pH = 2 | Buxton et al.,[5] |
| | 2.0 × $10^{7}$ | rec. avg. value | Buxton et al.,[5] |
| malonic acid anion | (6 ± 1) × $10^{7}$ | LFP, SCN$^-$-C.K., pH = 4.0, $k_{ref.}$ = 1.24 × $10^{10}$ L mol$^{-1}$ s$^{-1}$, A = (3.2 ± 0.4) × $10^{9}$ L mol$^{-1}$ s$^{-1}$, $E_A$ = 11 ± 5 kJ mol$^{-1}$ | Ervens et al.,[13] |
| malonate (di-anion) | (3.0 ± 1.6) × $10^{8}$ | LFP, SCN$^-$-C.K., pH = 6-7, $k_{ref.}$ = 1.24 × $10^{10}$ L mol$^{-1}$ s$^{-1}$ | Buxton et al.,[5] |

| reactant | $k_{2nd}$ / L mol$^{-1}$ s$^{-1}$ | remarks | references |
|---|---|---|---|
| tartronic acid | 1.7 × $10^8$ | PR, $SCN^-$-C.K., pH = 1, $k_{ref.}$ = 1.1 × $10^{10}$ L mol$^{-1}$ s$^{-1}$ | Schuchmann et al.,[20] |
| tartronic acid anion | 3.6 × $10^8$ | PR, $SCN^-$-C.K., pH = 3, $k_{ref.}$ = 1.1 × $10^{10}$ L mol$^{-1}$ s$^{-1}$ | Schuchmann et al.,[20] |
| tartronate | 4.4 × $10^8$ | PR, $SCN^-$-C.K., pH = 6-10, $k_{ref.}$ = 1.1 × $10^{10}$ L mol$^{-1}$ s$^{-1}$ | Schuchmann et al.,[20] |
| **methylmalonic acid** | **(1.6 ± 0.1) × $10^8$** | LFP, $SCN^-$-C.K., $k_{ref.}$ = 1.19 × $10^{10}$ L mol$^{-1}$ s$^{-1}$, pH = 1.0, A = (5.5 ± 0.1) × $10^{10}$ L mol$^{-1}$ s$^{-1}$, $E_A$ = 15 ± 1 kJ mol$^{-1}$ | **This study** |
| **methylmalonic acid anion** | **(2.3 ± 0.4) × $10^8$** | LFP, $SCN^-$-C.K., $k_{ref.}$ = 1.19 × $10^{10}$ L mol$^{-1}$ s$^{-1}$, pH = 4.4 A = (1.4 ± 0.1) × $10^9$ L mol$^{-1}$ s$^{-1}$, $E_A$ = 4 ± 1 kJ mol$^{-1}$ | **This study** |
| **methylmalonate (di-anion)** | **(6.2 ± 0.1) × $10^8$** | LFP, $SCN^-$-C.K., $k_{ref.}$ = 1.19 × $10^{10}$ L mol$^{-1}$ s$^{-1}$, pH = 8.5, A = (9.6 ± 0.4) × $10^{10}$ L mol$^{-1}$ s$^{-1}$, $E_A$ = 13 ± 1 kJ mol$^{-1}$ | **This study** |
| succinic acid | (1.3 ± 0.2) × $10^8$ | LFP, $SCN^-$-C.K., $k_{ref.}$ = 1.19 × $10^{10}$ L mol$^{-1}$ s$^{-1}$, pH = 2.0, A = (2.1 ± 0.1) × $10^{10}$ L mol$^{-1}$ s$^{-1}$, $E_A$ = 13 ± 2 kJ mol$^{-1}$ | Schaefer et al.,[21] |
| succinic acid anion | (4.6 ± 0.3) × $10^8$ | LFP, $SCN^-$-C.K., $k_{ref.}$ = 1.19 × $10^{10}$ L mol$^{-1}$ s$^{-1}$, pH = 4.9, A = (1.5 ± 0.1) × $10^{10}$ L mol$^{-1}$ s$^{-1}$, $E_A$ = 9 ± 3 kJ mol$^{-1}$ | Schaefer et al.,[21] |
| succinate (di-anion) | (5.3 ± 0.2) × $10^8$ | LFP, $SCN^-$-C.K., $k_{ref.}$ = 1.19 × $10^{10}$ L mol$^{-1}$ s$^{-1}$, pH = 8.0, A = (2.9 ± 0.2) × $10^{11}$ L mol$^{-1}$ s$^{-1}$, $E_A$ = 15 ± 3 kJ mol$^{-1}$ | Schaefer et al.,[21] |
| malic acid | (3.6 ± 1.6) × $10^8$ | LFP, $SCN^-$-C.K., pH = 1.0, $k_{ref.}$ = 1.24 × $10^{10}$ L mol$^{-1}$ s$^{-1}$, A = (7.9 ± 0.8) × $10^{10}$ L mol$^{-1}$ s$^{-1}$, $E_A$ = 13 ± 7 kJ mol$^{-1}$ | Gligorovski et al.,[22] |
| malic acid anion | (9.7 ± 2.5) × $10^8$ | LFP, $SCN^-$-C.K., pH = 4.3, $k_{ref.}$ = 1.24 × $10^{10}$ L mol$^{-1}$ s$^{-1}$, A = (2.9 ± 0.4) × $10^{11}$ L mol$^{-1}$ s$^{-1}$, $E_A$ = 14 ± 10 kJ mol$^{-1}$ | Gligorovski et al.,[22] |
| maleate (di-anion) | (8.5 ± 1.1) × $10^8$ | LFP, $SCN^-$-C.K., pH = 9, $k_{ref.}$ = 1.24 × $10^{10}$ L mol$^{-1}$ s$^{-1}$, A = (1.2 ± 0.2) × $10^{11}$ L mol$^{-1}$ s$^{-1}$, $E_A$ = 12 ± 10 kJ mol$^{-1}$ | Gligorovski et al.,[22] |
| tartaric acid | (3.6 ± 0.1) × $10^8$ | LFP, $SCN^-$-C.K., $k_{ref.}$ = 1.19 × $10^{10}$ L mol$^{-1}$ s$^{-1}$, pH = 1.0, A = (3.3 ± 0.1) × $10^{10}$ L mol$^{-1}$ s$^{-1}$, $E_A$ = 11 ± 1 kJ mol$^{-1}$ | Yang et al.,[23] |
| tartaric acid anion | (5.0 ± 0.1) × $10^8$ | LFP, $SCN^-$-C.K., $k_{ref.}$ = 1.19 × $10^{10}$ L mol$^{-1}$ s$^{-1}$, pH = 3.65, A = (3.6 ± 0.1) × $10^{10}$ L mol$^{-1}$ s$^{-1}$, $E_A$ = 5 ± 1 kJ mol$^{-1}$ | Yang et al.,[23] |
| tartarte (di-anion) | (6.2 ± 0.1) × $10^8$ | LFP, $SCN^-$-C.K., $k_{ref.}$ = 1.19 × $10^{10}$ L mol$^{-1}$ s$^{-1}$, pH = 7.0, A = (3.3 ± 0.1) × $10^{10}$ L mol$^{-1}$ s$^{-1}$, $E_A$ = 10 ± 1 kJ mol$^{-1}$ | Yang et al.,[23] |
| glutaric acid | (5.5 ± 0.1) × $10^8$ | LFP, $SCN^-$-C.K., $k_{ref.}$ = 1.19 × $10^{10}$ L mol$^{-1}$ s$^{-1}$, pH = 2.0, A = (3.9 ± 0.1) × $10^{10}$ L mol$^{-1}$ s$^{-1}$, $E_A$ = 11 ± 2 kJ mol$^{-1}$ | Wen et al.,[24] |
| glutaric acid anion | (8.6 ± 0.1) × $10^8$ | LFP, $SCN^-$-C.K., $k_{ref.}$ = 1.19 × $10^{10}$ L mol$^{-1}$ s$^{-1}$, pH = 4.9, A = (2.3 ± 0.1) × $10^{11}$ L mol$^{-1}$ s$^{-1}$, $E_A$ = 14 ± 2 kJ mol$^{-1}$ | Wen et al.,[24] |
| glutarate (di-anion) | (1.4 ± 0.1) × $10^9$ | LFP, $SCN^-$-C.K., $k_{ref.}$ = 1.19 × $10^{10}$ L mol$^{-1}$ s$^{-1}$, pH = 8.0, A = (1.4 ± 0.1) × $10^{11}$ L mol$^{-1}$ s$^{-1}$, $E_A$ = 12 ± 1 kJ mol$^{-1}$ | Wen et al.,[24] |

| reactant | $k_{2nd}$ / L mol$^{-1}$ s$^{-1}$ | remarks | references |
|---|---|---|---|
| adipic acid | $(1.4 \pm 0.1) \times 10^{9}$ | LFP, SCN$^-$-C.K., $k_{ref.} = 1.19 \times 10^{10}$ L mol$^{-1}$ s$^{-1}$, pH = 2.0, A = $(7.5 \pm 0.2) \times 10^{10}$ L mol$^{-1}$ s$^{-1}$, $E_A = 10 \pm 1$ kJ mol$^{-1}$ | Wen et al.,[24] |
| adipic acid anion | $(1.8 \pm 0.1) \times 10^{9}$ | LFP, SCN$^-$-C.K., $k_{ref.} = 1.19 \times 10^{10}$ L mol$^{-1}$ s$^{-1}$, pH = 4.9, A = $(9.5 \pm 0.3) \times 10^{10}$ L mol$^{-1}$ s$^{-1}$, $E_A = 10 \pm 2$ kJ mol$^{-1}$ | Wen et al.,[24] |
| adipate (di-anion) | $(2.0 \pm 0.1) \times 10^{9}$ | LFP, SCN$^-$-C.K., $k_{ref.} = 1.19 \times 10^{10}$ L mol$^{-1}$ s$^{-1}$, pH = 8.0, A = $(8.7 \pm 0.2) \times 10^{10}$ L mol$^{-1}$ s$^{-1}$, $E_A = 9 \pm 1$ kJ mol$^{-1}$ | Wen et al.,[24] |
| mucic acid or galactaric acid | $(4.5 \pm 0.1) \times 10^{8}$ | LFP, SCN$^-$-C.K., $k_{ref.} = 1.19 \times 10^{10}$ L mol$^{-1}$ s$^{-1}$, pH = 1.0, A = $(2.2 \pm 0.1) \times 10^{11}$ L mol$^{-1}$ s$^{-1}$, $E_A = 9 \pm 1$ kJ mol$^{-1}$ | Yang et al.,[23] |
| galactaric acid anion | $(6.9 \pm 0.3) \times 10^{8}$ | LFP, SCN$^-$-C.K., $k_{ref.} = 1.19 \times 10^{10}$ L mol$^{-1}$ s$^{-1}$, pH = 3.35, A = $(5.1 \pm 0.1) \times 10^{11}$ L mol$^{-1}$ s$^{-1}$, $E_A = 11 \pm 1$ kJ mol$^{-1}$ | Yang et al.,[23] |
| galactarte (di-anion) | $(7.9 \pm 0.1) \times 10^{8}$ | LFP, SCN$^-$-C.K., $k_{ref.} = 1.19 \times 10^{10}$ L mol$^{-1}$ s$^{-1}$, pH = 7.0, A = $(2.1 \pm 0.1) \times 10^{11}$ L mol$^{-1}$ s$^{-1}$, $E_A = 8 \pm 1$ kJ mol$^{-1}$ | Yang et al.,[23] |
| pimilic acid | $(2.2 \pm 0.1) \times 10^{9}$ | LFP, SCN$^-$-C.K., $k_{ref.} = 1.19 \times 10^{10}$ L mol$^{-1}$ s$^{-1}$, pH = 2.0, A = $(7.3 \pm 0.2) \times 10^{10}$ L mol$^{-1}$ s$^{-1}$, $E_A = 9 \pm 1$ kJ mol$^{-1}$ | Schaefer et al.,[21] |
| pimilic acid anion | $(3.4 \pm 0.3) \times 10^{9}$ | LFP, SCN$^-$-C.K., $k_{ref.} = 1.19 \times 10^{10}$ L mol$^{-1}$ s$^{-1}$, pH = 4.6, A = $(1.8 \pm 0.1) \times 10^{11}$ L mol$^{-1}$ s$^{-1}$, $E_A = 10 \pm 2$ kJ mol$^{-1}$ | Schaefer et al.,[21] |
| pimelate (di-anion) | $(3.2 \pm 0.1) \times 10^{9}$ | LFP, SCN$^-$-C.K., $k_{ref.} = 1.19 \times 10^{10}$ L mol$^{-1}$ s$^{-1}$, pH = 8.0, A = $(1.4 \pm 0.1) \times 10^{12}$ L mol$^{-1}$ s$^{-1}$, $E_A = 15 \pm 1$ kJ mol$^{-1}$ | Schaefer et al.,[21] |

LFP: laser flash photolysis, P.R.: pulse radiolysis, SCN$^-$-C.K.: thiocyanate competition kinetics, γ-R: γ-radiolysis, MeOH-C.K.: methanol reference reactant competition kinetics, EtOH-C.K.: ethanol reference reactant competition kinetics

# References


(1) Herrmann, H.; Hoffmann, D.; Schaefer, T.; Bräuer, P.; Tilgner, A. Tropospheric Aqueous-Phase Free-Radical Chemistry: Radical Sources, Spectra, Reaction Kinetics and Prediction Tools. *ChemPhysChem* **2010**, *11* (18), 3796-3822. DOI: 10.1002/cphc.201000533.

(2) Martell, A. E.; Smith, R. M. Carboxylic Acids. In *Critical Stability Constants: First Supplement*, Martell, A. E., Smith, R. M. Eds.; Springer US, 1982; pp 284-332.

(3) Silva, A. M. N.; Kong, X.; Hider, R. C. Determination of the pKa value of the hydroxyl group in the α-hydroxycarboxylates citrate, malate and lactate by 13C NMR: implications for metal coordination in biological systems. *BioMetals* **2009**, *22* (5), 771-778. DOI: 10.1007/s10534-009-9224-5.

(4) von Smoluchowski, M. Mathematical Theory of the Kinetics of the Coagulation of Colloidal Solutions. *Z. Phys. Chem.* **1917**, *92* (1), 129-168. DOI: 10.1515/zpch-1918-9209.

(5) Buxton, G. V.; Greenstock, C. L.; Helman, W. P.; Ross, A. B. Critical Review of rate constants for reactions of hydrated electrons, hydrogen atoms and hydroxyl radicals (·OH/·O⁻ in Aqueous Solution. *J. Phys. Chem. Ref. Data* **1988**, *17* (2), 513-886. DOI: 10.1063/1.555805.

(6) Hayduk, W.; Laudie, H. Prediction of diffusion coefficients for nonelectrolytes in dilute aqueous solutions. *AIChE Journal* **1974**, *20* (3), 611-615. DOI: 10.1002/aic.690200329.

(7) Kestin, J.; Sokolov, M.; Wakeham, W. A. Viscosity of Liquid Water in the Range - 8 C to 150 C. *J. Phys. Chem. Ref. Data* **1978**, *7*, 941-948. DOI: 10.1063/1.555581.

(8) Joback, K. G.; Reid, R. C. Estimation of pure component properties from group contributions. *Chem. Eng. Commun.* **1987**, *57* (1-6), 233-243. DOI: 10.1080/00986448708960487.

(9) Minakata, D.; Li, K.; Westerhoff, P.; Crittenden, J. Development of a Group Contribution Method To Predict Aqueous Phase Hydroxyl Radical ($HO^•$) Reaction Rate Constants. *Environ. Sci. Technol.* **2009**, *43* (16), 6220-6227. DOI: 10.1021/es900956c.

(10) Doussin, J. F.; Monod, A. Structure–activity relationship for the estimation of OH-oxidation rate constants of carbonyl compounds in the aqueous phase. *Atmos. Chem. Phys.* **2013**, *13* (23), 11625-11641. DOI: 10.5194/acp-13-11625-2013.

(11) Witkowski, B.; Jain, P.; Wileńska, B.; Gierczak, T. Temperature-dependent aqueous OH kinetics of C2-C10 linear and terpenoid alcohols and diols: new rate coefficients, structure-activity relationship and atmospheric lifetimes. *EGUsphere* **2023**, *2023*, 1-35. DOI: 10.5194/egusphere-2023-1381.

(12) Chin, M.; Wine, P. H. A temperature-dependent competitive kinetics study of the aqueous-phase reactions of OH radicals with formate, formic acid, acetate, acetic acid, and hydrated formaldehyde. In *Aquatic and Surface Photochemistry*, Helz, G., Zepp, G., Crosby, D. G. Eds.; Lewis, 1994; pp 85-96.

(13) Ervens, B.; Gligorovski, S.; Herrmann, H. Temperature-dependent rate constants for hydroxyl radical reactions with organic compounds in aqueous solutions. *Phys. Chem. Chem. Phys.* **2003**, *5* (9), 1811-1824, 10.1039/B300072A. DOI: 10.1039/B300072A.

(14) Bell, J. A.; Grunwald, E.; Hayon, E. Kinetics of deprotonation of organic free radicals in water. Reaction of glycolate ($HOCHCO_2^-$), ($HOCHCONH_2$), and ($HOCCH_3CONH_2$) with various bases. *JACS* **1975**, *97* (11), 2995-3000. DOI: 10.1021/ja00844a014.

(15) Logan, S. R. Redox reactions of organic radicals with ferrocene/ferricenium species in aqueous solution. Part 1. Radicals derived from carboxylic acids. *J. Chem. Soc. Perkin Trans.* **1989**, *2*, 751-754. DOI: 10.1039/P29890000751.

(16) Martin, L. R.; Mezyk, S. P.; Mincher, B. J. Determination of Arrhenius and Thermodynamic Parameters for the Aqueous Reaction of the Hydroxyl Radical with Lactic Acid. *J. Phys. Chem. A* **2009**, *113* (1), 141-145. DOI: 10.1021/jp806290s.

(17) Adams, G. E.; Boag, J. W.; Currant, J.; Michael, B. D. Absolute rate constants for the reaction of the hydroxyl radical with organic compounds. In *Pulse Radiolysis*, Ebert, M., Keene, J. P., Swallow, A. J., Baxendale, J. H. Eds.; Academic Press, 1965; pp 131 - 143.

(18) Verma, N. C.; Fessenden, R. W. Time resolved ESR spectroscopy. IV. Detailed measurement and analysis of the ESR time profile. *J. Chem. Phys.* **1976**, *65* (6), 2139-2155. DOI: 10.1063/1.433370.

(19) *IUPAC Task Group on Atmospheric Chemical Kinetic Data Evaluation*. http://iupac.pole-ether.fr (accessed October 2024).

(20) Schuchmann, M. N.; Schuchmann, H.-P.; von Sonntag, C. Oxidation of hydroxymalonic acid by OH radicals in the presence and in the absence of molecular oxygen. A pulse-radiolysis and product study. *J. Phys. Chem.* **1995**, *99* (22), 9122-9129.

(21) Schaefer, T.; Wen, L.; Estelmann, A.; Maak, J.; Herrmann, H. pH- and temperature-dependent kinetics of the oxidation reactions of OH with succinic and pimelic acid in aqueous solution. *Atmosphere* **2020**, *11* (4), 320-333. DOI: 10.3390/atmos11040320.

(22) Gligorovski, S.; Rousse, D.; George, C. H.; Herrmann, H. Rate constants for the OH reactions with oxygenated organic compounds in aqueous solution. *Int. J. Chem. Kinet.* **2009**, *41* (5), 309-326. DOI: 10.1002/kin.20405.

(23) Yang, D.; Schaefer, T.; Wen, L.; Herrmann, H. Temperature- and pH- Dependent OH Radical Reaction Kinetics of Tartaric and Mucic Acids in the Aqueous Phase. *J. Phys. Chem. A* **2022**, *126* (36), 6244-6252. DOI: 10.1021/acs.jpca.2c03044.
(24) Wen, L.; Schaefer, T.; Zhang, Y.; He, L.; Ventura, O. N.; Herrmann, H. T- and pH-dependent OH radical reaction kinetics with glycine, alanine, serine, and threonine in the aqueous phase. *Phys. Chem. Chem. Phys.* **2022**, *24* (18), 11054-11065. DOI: 10.1039/D1CP05186E.
